# Patterns and risk of cardiovascular disease in rheumatoid arthritis and psoriatic arthritis: a nationwide cohort study in the UK

**DOI:** 10.1093/rap/rkag016

**Published:** 2026-01-24

**Authors:** Zijing Yang, Fabiola Atzeni, Mark Russell, Kaiyang Song, Callum Coalwood, Elizabeth Price, Maya Buch, Sam Norton, James Galloway

**Affiliations:** Department of Inflammation Biology, Centre for Rheumatic Diseases, King’s College London, London, UK; Department of Experimental and Internal Medicine, University of Messina, Messina, Italy; Department of Inflammation Biology, Centre for Rheumatic Diseases, King’s College London, London, UK; Department of Inflammation Biology, Centre for Rheumatic Diseases, King’s College London, London, UK; National Early Inflammatory Autoimmune Diseases Audit, British Society for Rheumatology, London, UK; Department of Rheumatology, Great Western Hospital NHS Foundation Trust, Swindon, UK; Centre for Musculoskeletal Research, University of Manchester, Manchester, UK; Department of Inflammation Biology, Centre for Rheumatic Diseases, King’s College London, London, UK; Department of Psychology, Institute of Psychiatry, Psychology and Neuroscience, King’s College London, London, UK; Department of Inflammation Biology, Centre for Rheumatic Diseases, King’s College London, London, UK

**Keywords:** cardiovascular risk, rheumatoid arthritis, psoriatic arthritis

## Abstract

**Objective:**

Patients with inflammatory arthritis (IA), including RA and PsA, have an elevated cardiovascular disease (CVD) risk. It is unclear whether this persists in the modern treat-to-target era, particularly in early disease. This study evaluates CVD risk and mortality in a contemporary early RA and PsA cohort.

**Methods:**

Adults (≥18 years) with newly diagnosed RA or PsA registered in the National Early Inflammatory Arthritis Audit (NEIAA) from 2018 to 2023 were included. Incidence rates of CVD events, major adverse cardiovascular events (MACE) and all-cause and CVD mortality were calculated. Standardized incidence and mortality ratios compared outcomes with the general population. Competing risk regression assessed factors associated with CVD outcomes.

**Results:**

A total of 1012/17 669 RA and 104/3271 PsA participants had CVD hospitalizations. The incidence of MACE hospitalizations was 2.4/100 person-years (95% CI 2.2, 2.7) in RA and 0.6/100 person-years (95% CI 0.4, 1.0) in PsA. The risk of CVD hospitalizations was ≈25% higher in IA participants. Compared with the general population, all-cause mortality was 1.1 times higher in RA. CVD incidence and mortality were consistently higher in males. Hypertension, diabetes and smoking were associated with an increased CVD risk. Early corticosteroid use did not predict CVD, while better early RA treatment response was linked to lower CVD risk.

**Conclusion:**

A greater incidence of CVD and MACE exists in early IA, although less than previously reported. Traditional CVD risk factors are the dominant explanation for the risk increase, with disease control being a smaller but significant contributor to risk. Routine cardiovascular risk assessment in early IA remains justified.

Key messages

- A significant cardiovascular risk gap emerges early in IA, reinforcing the need for prompt assessment.
- Traditional cardiovascular risk factors dominate, but effective early disease control remains clinically important.
- Routine cardiovascular risk screening should be integrated into early RA and PsA management pathways.

## Introduction

There has long been a recognized association between inflammatory arthritis (IA) and cardiovascular risk, with both premature onset of cardiovascular disease (CVD) as well as more deleterious outcomes attributable to cardiac events [1–5]. This has been best described for RA, with a complex interplay being identified between shared risk factors such as smoking, a direct association between IA and vascular disease through altered endothelial biology and an association between the treatments used for IA and CVD, in particular chronic corticosteroid exposure [6, 7].

Comorbidities and traditional cardiovascular risk factors contribute to CVD risk in RA and PsA [7, 8]. In addition, RA-specific risk factors such as chronic inflammation, seropositivity and corticosteroid use are associated with accelerated atherosclerosis in RA patients [1, 2, 8]. Although the evidence of the association between PsA and increased CVD risk is less well-established compared with the RA population, PsA has a stronger association with metabolic syndrome than RA [9], and most studies point towards a CVD risk in PsA that is broadly similar to that observed in RA [10, 11].

With increasing awareness of cardiovascular risks in IA, the need for comprehensive assessment and management of cardiovascular risk was emphasized. Over the last 20 years, research has looked to quantify risks and disentangle the relative contribution of each component [12, 13]. RA is now incorporated into risk calculators for CVD and national and international guidelines advocate for proactively screening for cardiovascular risk factors in people with IA [14, 15]. However, there is inconsistency of the evidence and the strength of recommendations across conditions, with PsA currently supported by lower levels of evidence and weaker recommendations.

There is evidence that the incidence of CVD events, all-cause mortality and CVD-related mortality have declined in RA and PsA over recent decades [16–18]. This improvement is likely attributable in part to the focus on early and aggressive treatment, with better long-term suppression of inflammation [15, 19–21]. Corticosteroid therapy is now avoided other than for early induction therapy or managing flares. The result of changing therapeutic strategies needs to be considered alongside changes in background risk factors, with declining population levels of smoking in many countries [22] but, in contrast to this, increasing levels of obesity leading to higher levels of diabetes and hypertension [23, 24]. Consequently, it is unclear how generalizable the findings from historic studies looking at the association between IA, its treatments and cardiovascular outcomes are in current times.

The aim of this study was to explore these questions. We used a UK inception cohort, recruiting patients from 2018 onwards, with new-onset IA, with linkage to a national hospital record system to enable us to capture information on cardiovascular outcomes. We set out to describe the contemporary incidence of these events and compare it with the background general population. We also explored the relative contribution of traditional cardiovascular risk factors, disease severity markers and early treatment strategies, including the early use of corticosteroids.

## Methods

### Data source

We used data from the National Early Inflammatory Arthritis Audit (NEIAA), a mandatory clinical audit program in England and Wales, which collates data on individuals with new diagnoses of IA. Data were contributed by providers of rheumatology services, who were mandated to submit data on all patients newly referred with suspected early inflammatory arthritis (EIA). Both individual and hospital-level data were collected via an online portal, which requires mandatory fields to be completed and validates the accuracy of the submitted data. These data were integrated with Hospital Episodes Statistics (HES) and the Office of National Statistics (ONS) datasets, including vital statistics, cancer registry, medical visits and hospital discharge data. CVD mortality and morbidity data were ascertained through linkage to the HES and ONS.

### Study population

We included all patients >18 years of age enrolled in the NEIAA with a diagnosis of RA or PsA between 3 May 2018 and 1 May 2023. The classification of RA and PsA was based on the expert rheumatologists’ working diagnosis following the first rheumatology visit.

### Outcome

The primary outcome was first presentation of composite hospitalization endpoints, namely major adverse cardiac events (MACE), or hospitalization for any CVD, as the primary reason recorded in secondary care. Three-point MACE was defined as non-fatal myocardial infarction, non-fatal stroke and cardiovascular death. The secondary outcomes were all-cause mortality and mortality from CVD. CVD-related death was when the underlying cause of death listed in the death registration certificate was CVD. All outcomes were defined using validated International Classification of Diseases version 10 codes [25, 26] in published papers and diagnostic codes are listed in Supplementary Table S1. All admission outcomes were obtained from linked HES data and mortality outcomes from linked ONS data. Patients were followed from IA diagnosis date until the earliest date of death, outcomes of interest or end date of the study (1 May 2023).

### Covariates

We considered demographic and clinical risk factors for CVD outcomes, including age, gender (male or female), ethnicity (White, Black, Asian, Mixed and other ethnicities), index of multiple deprivation [IMD, stratified into quintiles, from 1 (most deprived) to 5 (least deprived)] [27], smoking status (non-smoker, ex-smoker, current smoker), comorbidities [prior history of cancer, diabetes mellitus, CVD (defined in the NEIAA as a previous heart attack, stroke or peripheral vascular), fracture, lung disease, depression], Rheumatic Disease Comorbidity Index [from 0 (lowest) to 9 (highest)] [28] and whether individuals were prescribed corticosteroids at the time of the first rheumatology visit. The IMD is the official measure of small-area relative deprivation in England and Wales, which combines a set of domains, including income, employment, health and disability, crime, barriers to housing and services, living environment and education, skills and training [27].

Furthermore, we assessed disease-specific factors, including CRP, ESR, RF and CCO antibody status, baseline 28-joint DAS (DAS28) and the EULAR response (3-month). The EULAR response was classified as a non-, moderate or good response, depending on the extent of change in the DAS28 from baseline and the level of disease activity reached at 3 months [29].

### Statistical analysis

Categorical variables were presented as frequency and percentage and continuous variables as median and interquartile range (IQR). Crude incidence rates were calculated by dividing the number of incident CVD and/or MACE hospitalizations by the number of person-years (pys) in the cohort. For each participant, follow-up time was measured from the date of diagnosis until the first occurrence of the outcome of interest, death or censoring at the end of the study period. Category-specific rates were computed separately for subgroups of age, gender and ethnicity. Age calculations were based on the diagnosis year, which is when patients became at risk. To enable comparisons between the RA or PsA population and the general population, age- and gender-standardized rates and mortality were computed by applying direct age and gender standardization to the population census from the ONS using 10-year age bands [30]. We assumed a Poisson distribution when estimating 95% CIs. The age structure of the population in 2022 was used as the reference and the age-standardized rate was calculated by weighting their respective age-specific rates to the age structure of the reference population [30].

The expected number of CVD hospitalizations and deaths was computed by multiplying the age-specific and sex-specific follow-up time with the corresponding incidence in the general population, obtained from the ONS for death events [31] and from the Global Burden of Disease (GBD) Study 2019 [32] for CVD events. The standardized mortality ratio (SMR) and standardized incidence ratio (SIR) were calculated as the ratio of the number of observed to expected cases. For the SMR and SIR, 95% CIs were calculated using an exact Poisson distribution method.

We used a 3-month delayed entry model to study the association between remission attainment at 3 months after diagnosis and CVD, since the EULAR response was assessed 3 months after diagnosis. Cox proportional hazards regression models were used to estimate all-cause mortality. We used competing risk regression models for CVD, MACE and CVD death with the Fine–Gray subdistribution method [33] and non-cardiovascular deaths were treated as competing risks.

Univariable regression models were applied to estimate the association between individual factors and defined outcomes. Age and gender were then included as covariates in adjusted models. We did not include further interactions as they were not of primary interest in this study. All analyses were conducted separately in RA and PsA.

Results were reported as hazard ratios (HRs) and adjusted HRs (aHRs), with 95% CIs. All statistical analyses were performed using RStudio version 4.4.1 (R Foundation for Statistical Computing, Vienna, Austria). All *P*-values were two-tailed and *P* < 0.05 indicated statistical significance. The study followed the Strengthening the Reporting of Observational Studies in Epidemiology guideline for cohort studies.

### Missing data

We performed multiple imputations using the chained equations method (*n* = 20) with the imputed datasets combined for analysis using Rubin’s formula. To address missing values we employed the predictive mean matching imputation method for the continuous variables (baseline DAS28, EULAR response in 3 months, CRP and ESR).

### Sensitivity analysis

We performed several sensitivity analyses to test the robustness of our results: we excluded participants with cancer in the analysis of mortality; since in the main analysis we considered hypertension and diabetes present if recorded at any time during follow-up, they were considered present at baseline in the sensitivity analysis; we repeated all analyses using a complete case approach; and we repeated all analyses in the patients without previous heart attack, stroke or peripheral vascular history.

### Ethical approval

No informed patient consent was required, as the NEIAA has permission from the Secretary of State for Health to collect data for the purposes of national audit. Ethical approval for the secondary use in the NEIAA has been obtained (Clinical Advisory Group Reference: 19/CAG/0059; Research Ethics Committee reference: 19/EE/0082).

## Results

### Characteristics

A total of 20 940 participants (17 669 in RA, 3271 in PsA) were identified within our study with a median age of 59 years [IQR 47–70; 61 (IQR 49–70) in RA, 48 (IQR 36–59) in PsA] and a female proportion of 62% [13 004; 63% (11 197) in RA, 55% (1807) in PsA]. Table 1 shows the baseline characteristics and detailed information is presented in Supplementary Table S2.

**Table 1:** Baseline data.

Characteristics	Overall (*N* = 20 940	RA (*N* = 17 669)	PsA (*N* = 3271)
Age of diagnosis, years, median (IQR)	59 (47–70)	61 (49–72)	48 (36–59)
Age of diagnosis, years, *n* (%)			
18–39	3224 (15.4)	2146 (12.1)	1078 (33)
40–49	2942 (14)	2275 (13)	667 (20)
50–59	4638 (22)	3871 (22)	767 (23)
60–69	4557 (22)	4122 (23)	435 (13)
70–79	4108 (20)	3823 (22)	285 (8.7)
≥80	1471 (7.0)	1432 (8.1)	39 (1.2)
Gender, *n* (%)			
Female	13 004 (62)	11 197 (63)	1807 (55)
Male	7936 (38)	6472 (37)	1464 (45)
Ethnicity, *n* (%)			
White	17 836 (85)	14 957 (85)	2879 (88)
Asian	1598 (7.6)	1381 (7.8)	217 (6.6)
Black	498 (2.4)	471 (2.7)	27 (0.8)
Other	780 (3.7)	670 (3.8)	110 (3.4)
Smoking status, *n* (%)			
Never smoked	9068 (43)	7539 (43)	1529 (47)
Current smoker	3928 (19)	3374 (19)	554 (17)
Ex-smoker	5955 (28)	5124 (29)	831 (25)
Comorbidity, *n* (%)			
Lung disease	2134 (10)	1991 (11)	143 (4.4)
Heart attack	1127 (5.4)	1027 (5.8)	100 (3.1)
Hypertension	4767 (23)	4295 (24)	472 (14)
Diabetes	2071 (9.9)	1834 (10)	237 (7.2)
Cancer	812 (3.9)	729 (4.1)	83 (2.5)
Stomach ulcer	708 (3.4)	628 (3.6)	80 (2.4)
Depression	1609 (7.7)	1292 (7.3)	317 (9.7)
Seropositive, n/N (%)	12 331 (59)	12 041 (68)	290 (8.9)
Baseline corticosteroids, n/N (%)	15 280 (73)	13 638 (77)	1642 (50)
First-line csDMARD treatment, *n* (%)			
Baseline DAS28, *n* (%)			
Low	2900 (14)	2224 (13)	676 (21)
Moderate	8139 (39)	6707 (38)	1432 (44)
High	8534 (41)	7767 (44)	767 (23)
Duration of symptoms (months), *n* (%)			
<3	8262 (40)	7388 (42)	874 (27)
3–6	4932 (24)	4189 (24)	743 (23)
6–12	3977 (19)	3255 (18)	722 (22)
>12	3610 (17)	2712 (15)	898 (27)
EULAR response (3-month), *n* (%)			
No response	4478 (21)	3756 (21)	722 (22)
Good response	4798 (23)	4345 (25)	453 (14)
Moderate response	3980 (19)	3509 (20)	471 (14)
CCP positive, *n* (%)	9478 (45)	9362 (53)	116 (3.5)
CRP, mg/dl, median (IQR)	11 (4–28)	11 (4–29)	7 (3–19)
Elevation, *n* (%)	18 233 (87)	15 543 (88)	2690 (82)
ESR, mm/h, median (IQR)	26 (11–44)	27 (12–45)	16 (6–33)
High, *n* (%)	7018 (34)	6199 (35)	819 (25)

### CVD incidence

RA and PsA participants were followed for a median of 2.45 years (IQR 1.1–4.0) and 2.53 years (IQR 1.2–4.1), respectively, contributing a total of 44 968 and 8541 pys at risk. Over this period, 1012 CVD events [5.7% (95% CI 5.4, 6.1); age and gender standardized: 4.2%] occurred among the RA participants and 104 CVD events [3.2% (95% CI 2.6, 3.8); age and gender standardized: 3.8%] the among PsA participants. The incidence of CVD hospitalizations was 2.25/100 pys (95% CI 2.11, 2.39) in RA and 1.2/100 pys (95% CI 9.9, 1.5) in PsA. The incidences of MACE among RA and PsA participants are shown in Table 2. More information on stratified incidence by age and gender is provided in Supplementary Table S2 and Fig. S1.

**Table 2:** Incidence of cardiovascular events, MACE, all-cause death and CVD-related death.

Variables	CVD (n)	Proportion per 100 people, % (95% CI)	Incidence per 100 pys (95% CI)	MACE, *n*	Proportion per 100 people (95% CI)	Incidence per 100 pys (95% CI)
RA	1012	5.7 (5.4, 6.1)	2.3 (2.1, 2.4)	428	2.4 (2.2, 2.7)	1.0 (0.9, 1.1)
Female	514	4.6 (4.2, 5.0)	1.7 (1.6, 1.9)	204	1.8 (1.6, 2.1)	0.7 (0.6, 0.8)
Male	498	7.7 (7.1, 8.4)	3.1 (2.8, 3.4)	224	3.5 (3.0, 4.0)	1.3 (1.1, 1.5)
PsA	104	3.2 (2.6, 3.8)	1.2 (1.0, 1.5)	21	0.6 (0.4, 1.0)	0.2 (0.1, 0.4)
Female	41	2.3 (1.6, 3.1)	0.9 (0.6, 1.1)	7	0.4 (0.2, 0.8)	0.1 (0.06, 0.3)
Male	63	4.3 (3.3, 5.5)	1.7 (1.3, 2.1)	14	1.0 (0.05, 1.6)	0.4 (0.2, 0.6)

**Figure 1:**
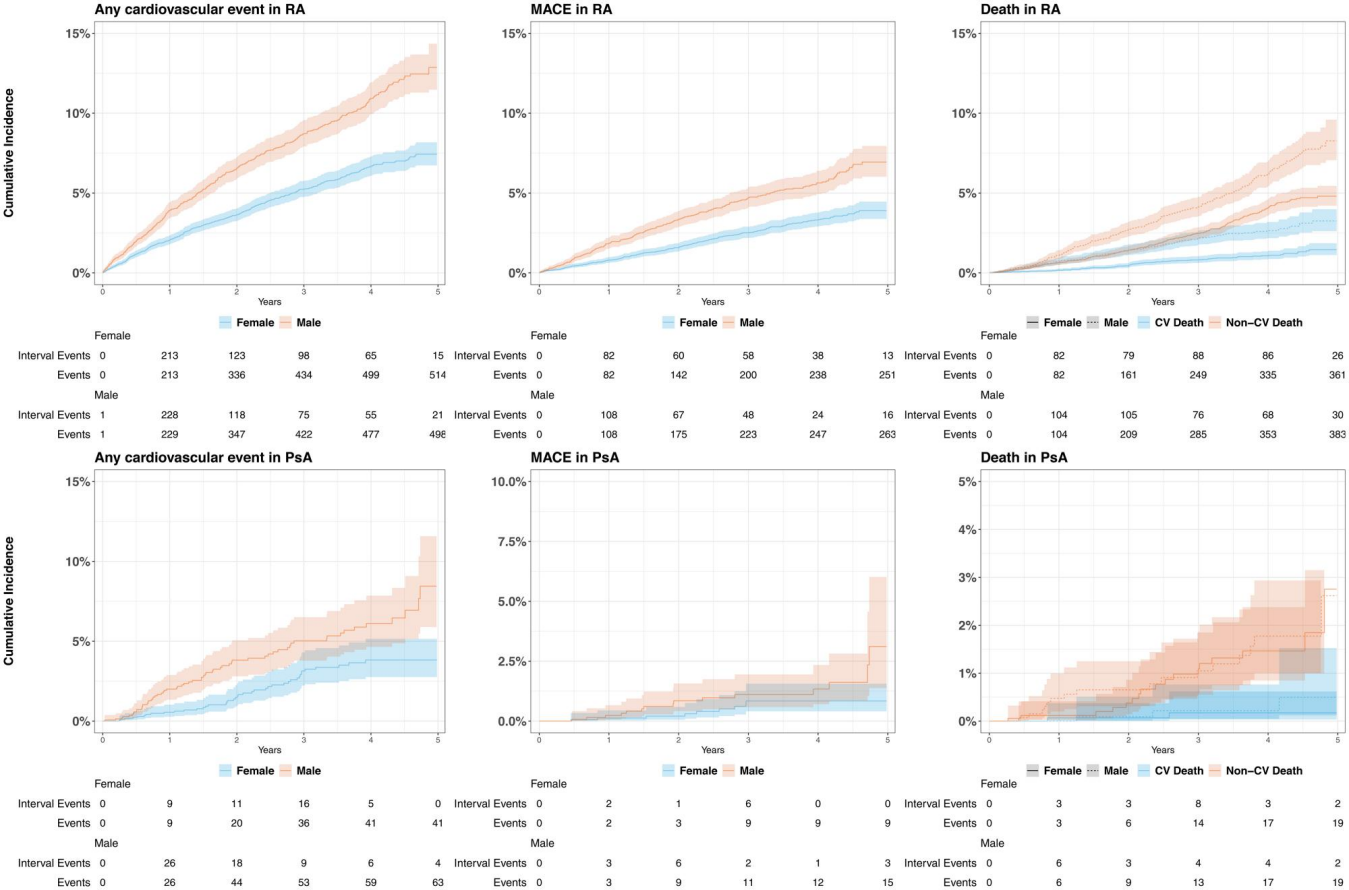
Cumulative incidence of outcomes in RA and PsA adjusted for age and stratified by sex

The unadjusted 5-year cumulative incidence of CVD and MACE in RA participants was 9.4% (95% CI 8.7, 10.1) and 5% (95% CI 4.5, 5.5), respectively, and 6.0% (95% CI 4.6, 7.4) and 1.9% (95% CI 1.0, 3.2), respectively, in PsA participants. A higher cumulative incidence of CVD and MACE was observed in males [CVD 12.9% (95% CI 11.5, 14.3); MACE 6.9% (95% CI 6.0, 8.0)] compared with females [CVD 7.4% (95% CI 6.7, 8.2); MACE 3.9% (95% CI 3.4, 4.5)]; with similar observations in PsA [males: CVD 8.5% (95% CI 6.0, 11.6), MACE 3.4% (95% CI 1.4, 6.0); females: CVD 3.8% (95% CI 2.8, 5.2), MACE 0.8% (95% CI 0.4, 1.6)]. Fig. 1 and Supplementary Fig. S2–4 illustrate the cumulative incidence of all outcomes of interest by gender. The incidence of each subgroup, such as RA and PsA without myocardial infarction history, is reported in Supplementary Table S3.

There was an overall 25% higher risk of CVD in RA compared with the general population [SIR 1.25 (95% CI 1.18, 1.33)] and 26% in PsA [SIR 1.26 (95% CI 1.03, 1.53)]. Males with RA had a higher SIR compared with males in the general population [1.34 (95% CI 1.22, 1.46)] relative to females with RA compared with females in the general population [1.18 (95% CI 1.08, 1.29)]. Among those <65 years of age with RA, the risks in females [1.32 (95% CI 1.13, 1.54)] and males [1.31 (95% CI 1.11, 1.54)] were similar, and both were higher than the overall risk [1.25 (95% CI 1.18, 1.33)]. A similar trend [overall: male 1.46 (95% CI 1.13, 1.87), female 1.02 (95% CI 0.74, 1.39); <65 years of age: male 1.59 (95% CI 1.13, 2.16), female: 1.52 (95% CI 1.02, 2.19)] was observed in the PsA population; more information is reported in Supplementary Tables S4 and S5.

### All-cause and CVD-related mortality

The unadjusted cumulative all-cause mortality during the study period was 8.2% (95% CI 7.5–8.9) in RA and 3.0% (95% CI 1.8–4.7) in PsA. The CVD-related cumulative mortality in RA [2.1% (95% CI 1.8, 2.5)] was considerably higher than in PsA [0.3% (95% CI 0.1, 0.7)]. Fig. 1 shows that the higher cumulative all-cause and CVD-related mortality in males was found both in PsA and RA. More information on the cumulative mortality in PsA and RA is provided in Supplementary Table S1 and Fig. S5. The all-cause SMR in RA compared with the general population was 1.11 (95% CI 1.03, 1.19), with male individuals having higher mortality [1.13 (95% CI 1.02, 1.25)] than female individuals [1.08 (95% CI 0.97, 1.19)] (Table 3 and Supplementary Table S3).

**Table 3:** Incidence of all-cause death and CVD-related death.

Variables	All-cause death (*n*)	Proportion per 100 people (95% CI)	Incidence per 100 pys (95% CI)	CVD death (*n*)	Proportion per 100 people (95% CI)	Incidence per 100 pys (95% CI)
RA	744	4.2 (3.9, 4.5)	1.6 (1.5, 1.7)	201	1.1 (1.0, 1.3)	0.4 (0.3, 0.5)
Female	361	3.2 (3.0, 3.6)	1.2 (1.1, 1.3)	82	0.7 (0.6, 0.9)	0.3 (0.2, 0.3)
Male	383	6.0 (5.4, 6.5)	2.2 (2.0, 2.5)	119	1.8 (1.5, 2.2)	0.7 (0.6, 0.8)
PsA	38	1.1 (0.8, 1.6)	0.4 (0.3, 0.6)	5	0.2 (0.05, 0.4)	0.06 (0.02, 0.1)
Female	19	1.0 (0.6, 1.6)	0.4 (0.2, 0.6)	2	0.1 (0.01, 0.4)	0.04 (0.007, 0.1)
Male	19	1.3 (0.8, 2.0)	0.5 (0.3, 0.8)	3	0.2 (0.04, 0.6)	0.08 (0.02, 0.2)

### Risk factors

After adjusting for age and gender, several traditional cardiovascular risk factors were significantly associated with CVD events in RA: current smoking [aHR 1.67 (95% CI 1.40, 1.99), *P* < 0.001], hypertension [aHR 1.51 (95% CI 1.32, 1.73), *P* < 0.001] and diabetes [aHR 1.44 (95% CI 1.22, 1.70), *P* < 0.001]. The association between smoking and CVD death was even more pronounced [aHR 3.08 (95% CI 2.10, 4.50), *P* < 0.001)]. Similar associations with these risk factors were observed in PsA (Table 4). Patients with a high DAS28 baseline score, elevated CRP and high ESR exhibited a numerically increased risk of CVD hospitalization without statistical significance (Supplementary Table S3). Moreover, there is no significant evidence showing the interaction of ESR/CRP with traditional CVD risk factors (smoking status, hypertension and diabetes) on CVD hospitalization. Patients who were positive for CCP or RF were more likely to develop MACE [1.49 (95% CI 1.21, 1.82), *P* < 0.001 and 1.36 (95% CI 1.12, 1.66), *P* = 0.002, respectively]. Patients with better treatment response to DMARDs at 3 months following an IA diagnosis had a decreased CVD risk [those with good treatment response: 0.80 (95% CI 0.69, 0.93), *P* = 0.004 for any CVD events; 0.75 (95% CI 0.6, 0.93), *P* = 0.009 for MACE; 0.58 (95% CI 0.41, 0.82), *P* = 0.002 for CVD death relative to no treatment response]. There was no significant association observed between depression and CVD and MACE in either the RA or PsA populations. There is no significant protective association with corticosteroids treatment as the initial induction therapy [1.00 (95% CI 0.85, 1.17)]. Full association information is reported in Table 4 and Supplementary Figs S6 and S7. Estimates using the complete-case method were similar (Supplementary Tables S6 and S7).

**Table 4:** Association of risk factors and CVD in RA.

Variable/outcome	RA (aHR), median (95% CI)			
	Any CVD	MACE	All-cause death	CVD death
Smoking status				
Never smoking (ref)				
Current smoker	1.67 (1.40, 1.99)	2.81 (2.17, 3.65)	3.11 (2.52, 3.85)	3.08 (2.10, 4.50)
Ex-smoker	1.29 (1.11, 1.50)	1.36 (1.06, 1.74)	1.63 (1.36, 1.95)	1.26 (0.88, 1.80)
Comorbidity				
Lung disease	1.49 (1.28, 1.75)	1.60 (1.27, 2.01)	2.26 (1.92, 2.65)	2.14 (1.58, 2.91)
Heart attack history	1.94 (1.62, 2.31)	1.80 (1.38, 2.34)	1.73 (1.42, 2.10)	1.76 (1.22, 2.54)
Hypertension	1.51 (1.32, 1.73)	1.43 (1.17, 1.74)	1.17 (1.01, 1.36)	1.38 (1.03, 1.85)
Diabetes	1.44 (1.22, 1.70)	1.64 (1.29, 2.08)	1.59 (1.33, 1.91)	1.91 (1.38, 2.66)
DAS28 baseline				
Low (ref)				
High	1.13 (0.92, 1.38)	1.27 (0.94, 1.72)	1.39 (1.07, 1.8)	1.26 (0.79, 2.01)
Moderate	0.95 (0.77, 1.18)	1.06 (0.78, 1.46)	1.11 (0.84, 1.45)	0.89 (0.49, 1.31)
RF positive	1.07 (0.94, 1.24)	1.36 (1.12, 1.66)	1.80 (1.51, 2.14)	1.89 (1.35, 2.65)
Elevation CRP baseline	1.19 (0.7, 2.01)	1.28 (0.59, 2.77)	1.67 (0.67, 4.14)	1.34 (0.35, 5.07)
High ESR baseline	1.01 (0.88, 1.15)	1.01 (0.83, 1.23)	1.39 (1.17, 1.65)	1.31 (0.94, 1.81)
CCP positive	1.08 (0.94, 1.25)	1.49 (1.21, 1.82)	1.75 (1.47, 2.08)	2.09 (1.48, 2.95)
EULAR response 3 month				
No response (ref)				
Good response	0.80 (0.69, 0.93)	0.75 (0.6, 0.93)	0.53 (0.44, 0.64)	0.58 (0.41, 0.82)
Moderate response	0.94 (0.8, 1.1)	0.87 (0.68, 1.11)	0.75 (0.62, 0.9)	0.71 (0.49, 1.03)

## Discussion

Our study presented an analysis of mortality and cardiovascular outcomes in patients with recently diagnosed RA or PsA and confirmed a significant CVD risk gap persists with the general population, albeit smaller than in previous studies. Crucially, our findings demonstrate the overriding importance of traditional cardiovascular risk factors, which emerged as dominant contributors to cardiovascular disease and mortality in this contemporary cohort. However, we also describe a clinically important and modifiable influence of inflammation, suggesting that effective disease control could reduce the risk of cardiovascular events.

Our observation of elevated CVD risk within a median follow-up of only 2.5 years after diagnosis represents an important novel finding. Historically, studies have reported elevated cardiovascular risks over extended periods. Our findings strongly reinforce the importance of immediate clinical attention to cardiovascular risk at the point of diagnosis rather than deferring management to later disease stages. The incidence of MACE in PsA was comparable to another study reporting an incidence of 0.29/100 pys [34] but is lower than in other studies [35, 36]. This discrepancy in PsA may be due to the different definitions of MACE across studies, the older median age at diagnosis and the higher proportion of males. We should note that even though more studies provided information about the CVD risk in PsA, the overall body of evidence remains limited. Additionally, we observed a higher incidence of CVD and MACE in early RA compared with early PsA, in line with results in most, but not all, previous studies [35–37]. The inclusion of an early PsA cohort in this analysis provides valuable insights into cardiovascular risk among a population that has historically been less thoroughly investigated [3, 38]. Although the sample size of the PsA cohort was smaller than that of the RA cohort, the data demonstrated a measurable increase in cardiovascular risk and death risk within the first few years of diagnosis. This early heightened cardiovascular risk in PsA has not been previously well characterized.

The incidence of MACE among RA patients in this study was slightly lower than that reported in the literature for the RA population from 1994 to 2010 in the UK (1.21/100 pys) [35]. Also, our study revealed a slight decrease in mortality for early RA patients compared with previous studies [4, 16, 39]. These decreases may reflect improvements in early RA management and an increased emphasis on early and aggressive therapeutic interventions. For example, guidelines encouraged MTX, especially MTX monotherapy as first-line treatment, and should be prescribed within 3 months for patients with RA [40]. In our cohort, 88% of RA patients were prescribed conventional synthetic DMARDs in the first 3 months after diagnosis, with 71% of them receiving MTX treatment. Additionally, broader public health measures targeting traditional cardiovascular risk factors, such as smoking cessation programs, may have contributed to narrowing the gap in cardiovascular outcomes between PA patients and the general population. However, this encouraging trend may be counterbalanced or even reversed in the future by rising obesity rates, which drive an increasing prevalence of hypertension, diabetes and metabolic syndrome, key factors that are particularly relevant to PsA [24, 41].

Previous studies have suggested increased cardiovascular risk associated with long-term corticosteroid exposure [42]. However, our study did not identify a significant association between corticosteroid prescription at diagnosis and subsequent cardiovascular risk. Possible reasons for the inconsistency could be that our analysis was limited to baseline corticosteroid prescription data rather than long-term use, and most patients received corticosteroids only for initial induction therapy or acute flare management. Furthermore, early initiation of DMARD therapy was strongly recommended, so we need to consider that improving systemic inflammation with concurrent DMARD use may mitigate the potential negative cardiovascular effects of corticosteroids.

Our study clearly demonstrated that traditional cardiovascular risk factors such as hypertension, diabetes and smoking dominate the overall cardiovascular risk burden, which should inform clinical prioritisation. Inflammation remains an important and modifiable risk factor that likely contributes to the persistent elevation of CVD risk, underscoring the need for cardiovascular prevention to be integrated into early IA management. The evidence on the significance of these factors as promoters of CVD in EIA was scarce and relies on composite definitions of endpoints. The average baseline CRP in our cohort was relatively low at 1.1 mg/l, which may obscure an association between inflammation and CVD risk. Importantly, our findings suggest that patients with good treatment responses within the first 3 months of diagnosis have a lower risk of CVD, which is consistent with the existing literature suggesting that achieving early and effective control of disease activity can reduce CVD risk [43, 44]. We acknowledge the complexity of attributing cardiovascular risk clearly to inflammation, medication use or traditional cardiovascular risk factors. Corresponding to findings in previous studies, the increased risk of CVD and death among people with RA or PsA remains likely to relate to suboptimal control of traditional cardiovascular risk factors [45]. Although baseline biomarker assessment was limited primarily to CRP and ESR, future research incorporating longitudinal measures of inflammation and detailed medication use over time is essential. In addition, more prospective mechanistic and causal inference studies in the future could better elucidate the complex interactions underpinning CVD risk.

Given the elevated cardiovascular risk identified very early in the disease course, our study emphasizes the importance of routine cardiovascular screening and aggressive modification of CVD risk factors at the time IA is diagnosed. The existing general population CVD risk prediction models mostly underestimated the risk [13] and the predictive performance of some validated RA-specific CVD risk algorithms appears suboptimal [12, 46]. Our data suggest incorporating serological status and early treatment response into clinical risk stratification algorithms to identify individuals who may require earlier and more aggressive cardiovascular preventive strategies. Early integration of these stratification measures could substantially improve the clinical outcomes of patients diagnosed with EIA.

### Strengths and limitations

We utilized one of the largest inception cohorts of early RA and PsA to provide an updated assessment of CVD risk. Some limitations of this study should be acknowledged. First, we could not obtain detailed medical data on other well-known risk factors for CVD, such as obesity and hyperlipidaemia. Second, the development of some chronic cardiovascular complications and death may necessitate a longer time frame to manifest and other mortality patterns might be observed with longer follow-up. As such, the median follow-up duration of 2.5 years may not have been sufficient to assess CVD and death comprehensively. Third, we were unable to examine cardiovascular risk in different ethnicities or age subgroups >80 years and <40 years due to small sample sizes and the low prevalence of outcomes. Furthermore, although we followed individuals longitudinally, outcomes were defined based on hospitalization data and primary care data were unavailable in this study. Therefore, some cases of CVD diagnosed in primary care were missed in our study. In addition, expected CVD events were estimated using GBD 2019 incidence rates, which do not account for the changes in cardiovascular incidence observed during and after the COVID-19 pandemic. These pandemic-related shifts, including interruptions to preventive care and changes in population structure, should be considered when interpreting the risk estimates. Also, in our analysis of treatment response we used a delayed entry model to assess the association between 3-month EULAR response and cardiovascular outcomes. This excluded individuals who experienced a CVD event or died within the first 3 months post-diagnosis (≈10% of all events), potentially introducing selection bias and limiting generalizability to patients who remained event-free during this early period. Considering these together, our results are likely to be an underestimate of the overall burden of CVD in EIA.

## Conclusion

Patients with early IA remain at elevated risk for CVD, MACE, all-cause mortality and CVD mortality compared with the general population. Effective disease control and management of traditional CVD risk factors remain core strategies for reducing overall risk. Given that cardiovascular risk factors are currently underidentified and managed in patients with EIA, our study calls for strategies to reduce cardiovascular risk as early as possible to become a routine part of early RA and PsA management.

## Supplementary Material

rkag016_Supplementary_Data

## Data Availability

Data from the NEIAA that were used to produce this analysis are available upon request, subject to approval of the Healthcare Quality Improvement Partnership and the BSR.
