# Supplementary material for: Patterns and risk of cardiovascular disease in rheumatoid arthritis and psoriatic arthritis: a nationwide cohort study in the UK
Source: Rheumatol Adv Pract. 2026 Jan 24;10(1):rkag016. doi: 10.1093/rap/rkag016 (PMC12900542; doi:10.1093/rap/rkag016)
Supplement: rkag016_Supplementary_Data [file rkag016_supplementary_data.docx]

Supplementary Table S1 ICD-10 code for CV definition

| **code** | **term** |
| --- | --- |
| I01 | Rheumatic fever with heart involvement |
| I010 | Acute rheumatic pericarditis |
| I011 | Acute rheumatic endocarditis |
| I012 | Acute rheumatic myocarditis |
| I018 | Other acute rheumatic heart disease |
| I019 | Acute rheumatic heart disease, unspecified |
| I020 | Rheumatic chorea with heart involvement |
| I029 | Rheumatic chorea without heart involvement |
| I05 | Rheumatic mitral valve diseases |
| I050 | Mitral stenosis |
| I051 | Rheumatic mitral insufficiency |
| I052 | Mitral stenosis with insufficiency |
| I058 | Other mitral valve diseases |
| I059 | Mitral valve disease, unspecified |
| I06 | Rheumatic aortic valve diseases |
| I060 | Rheumatic aortic stenosis |
| I061 | Rheumatic aortic insufficiency |
| I062 | Rheumatic aortic stenosis with insufficiency |
| I068 | Other rheumatic aortic valve diseases |
| I069 | Rheumatic aortic valve disease, unspecified |
| I07 | Rheumatic tricuspid valve diseases |
| I070 | Tricuspid stenosis |
| I071 | Tricuspid insufficiency |
| I072 | Tricuspid stenosis with insufficiency |
| I078 | Other tricuspid valve diseases |
| I079 | Tricuspid valve disease, unspecified |
| I08 | Multiple valve diseases |
| I080 | Disorders of both mitral and aortic valves |
| I081 | Disorders of both mitral and tricuspid valves |
| I082 | Disorders of both aortic and tricuspid valves |
| I083 | Combined disorders of mitral, aortic and tricuspid valves |
| I088 | Other multiple valve diseases |
| I089 | Multiple valve disease, unspecified |
| I09 | Other rheumatic heart diseases |
| I090 | Rheumatic myocarditis |
| I091 | Rheumatic diseases of endocardium, valve unspecified |
| I092 | Chronic rheumatic pericarditis |
| I098 | Other specified rheumatic heart diseases |
| I099 | Rheumatic heart disease, unspecified |
| I10 | Essential (primary) hypertension |
| I11 | Hypertensive heart disease |
| I110 | Hypertensive heart disease with (congestive) heart failure |
| I119 | Hypertensive heart disease without (congestive) heart failure |
| I13 | Hypertensive heart and renal disease |
| I130 | Hypertensive heart and renal disease with (congestive) heart failure |
| I131 | Hypertensive heart and renal disease with renal failure |
| I132 | Hypertensive heart and renal disease with both (congestive) heart failure and renal failure |
| I139 | Hypertensive heart and renal disease, unspecified |
| I20 | Angina pectoris |
| I200 | Unstable angina |
| I201 | Angina pectoris with documented spasm |
| I208 | Other forms of angina pectoris |
| I209 | Angina pectoris, unspecified |
| I21 | Acute myocardial infarction |
| I210 | Acute transmural myocardial infarction of anterior wall |
| I211 | Acute transmural myocardial infarction of inferior wall |
| I212 | Acute transmural myocardial infarction of other sites |
| I213 | Acute transmural myocardial infarction of unspecified site |
| I214 | Acute subendocardial myocardial infarction |
| I219 | Acute myocardial infarction, unspecified |
| I22 | Subsequent myocardial infarction |
| I220 | Subsequent myocardial infarction of anterior wall |
| I221 | Subsequent myocardial infarction of inferior wall |
| I228 | Subsequent myocardial infarction of other sites |
| I229 | Subsequent myocardial infarction of unspecified site |
| I23 | Certain current complications following acute myocardial infarction |
| I230 | Haemopericardium as current complication following acute myocardial infarction |
| I231 | Atrial septal defect as current complication following acute myocardial infarction |
| I232 | Ventricular septal defect as current complication following acute myocardial infarction |
| I233 | Rupture of cardiac wall without haemopericardium as current complication following acute myocardial infarction |
| I234 | Rupture of chordae tendineae as current complication following acute myocardial infarction |
| I235 | Rupture of papillary muscle as current complication following acute myocardial infarction |
| I236 | Thrombosis of atrium, auricular appendage, and ventricle as current complications following acute myocardial infarction |
| I238 | Other current complications following acute myocardial infarction |
| I24 | Other acute ischaemic heart diseases |
| I240 | Coronary thrombosis not resulting in myocardial infarction |
| I241 | Dressler syndrome |
| I248 | Other forms of acute ischaemic heart disease |
| I249 | Acute ischaemic heart disease, unspecified |
| I25 | Chronic ischaemic heart disease |
| I250 | Atherosclerotic cardiovascular disease, so described |
| I251 | Atherosclerotic heart disease |
| I252 | Old myocardial infarction |
| I253 | Aneurysm of heart |
| I254 | Coronary artery aneurysm and dissection |
| I255 | Ischaemic cardiomyopathy |
| I256 | Silent myocardial ischaemia |
| I258 | Other forms of chronic ischaemic heart disease |
| I259 | Chronic ischaemic heart disease, unspecified |
| I26 | Pulmonary embolism |
| I260 | Pulmonary embolism with mention of acute cor pulmonale |
| I269 | Pulmonary embolism without mention of acute cor pulmonale |
| I27 | Other pulmonary heart diseases |
| I270 | Primary pulmonary hypertension |
| I271 | Kyphoscoliotic heart disease |
| I272 | Other secondary pulmonary hypertension |
| I278 | Other specified pulmonary heart diseases |
| I279 | Pulmonary heart disease, unspecified |
| I28 | Other diseases of pulmonary vessels |
| I280 | Arteriovenous fistula of pulmonary vessels |
| I281 | Aneurysm of pulmonary artery |
| I288 | Other specified diseases of pulmonary vessels |
| I289 | Disease of pulmonary vessels, unspecified |
| I30 | Acute pericarditis |
| I300 | Acute nonspecific idiopathic pericarditis |
| I301 | Infective pericarditis |
| I308 | Other forms of acute pericarditis |
| I309 | Acute pericarditis, unspecified |
| I31 | Other diseases of pericardium |
| I310 | Chronic adhesive pericarditis |
| I311 | Chronic constrictive pericarditis |
| I312 | Haemopericardium, not elsewhere classified |
| I313 | Pericardial effusion (noninflammatory) |
| I318 | Other specified diseases of pericardium |
| I319 | Disease of pericardium, unspecified |
| I32 | Pericarditis in diseases classified elsewhere |
| I320 | Pericarditis in bacterial diseases classified elsewhere |
| I321 | Pericarditis in other infectious and parasitic diseases classified elsewhere |
| I328 | Pericarditis in other diseases classified elsewhere |
| I33 | Acute and subacute endocarditis |
| I330 | Acute and subacute infective endocarditis |
| I339 | Acute endocarditis, unspecified |
| I34 | Nonrheumatic mitral valve disorders |
| I340 | Mitral (valve) insufficiency |
| I341 | Mitral (valve) prolapse |
| I342 | Nonrheumatic mitral (valve) stenosis |
| I348 | Other nonrheumatic mitral valve disorders |
| I349 | Nonrheumatic mitral valve disorder, unspecified |
| I35 | Nonrheumatic aortic valve disorders |
| I350 | Aortic (valve) stenosis |
| I351 | Aortic (valve) insufficiency |
| I352 | Aortic (valve) stenosis with insufficiency |
| I358 | Other aortic valve disorders |
| I359 | Aortic valve disorder, unspecified |
| I37 | Pulmonary valve disorders |
| I370 | Pulmonary valve stenosis |
| I371 | Pulmonary valve insufficiency |
| I372 | Pulmonary valve stenosis with insufficiency |
| I378 | Other pulmonary valve disorders |
| I379 | Pulmonary valve disorder, unspecified |
| I38 | Endocarditis, valve unspecified |
| I39 | Endocarditis and heart valve disorders in diseases classified elsewhere |
| I390 | Mitral valve disorders in diseases classified elsewhere |
| I391 | Aortic valve disorders in diseases classified elsewhere |
| I392 | Tricuspid valve disorders in diseases classified elsewhere |
| I393 | Pulmonary valve disorders in diseases classified elsewhere |
| I394 | Multiple valve disorders in diseases classified elsewhere |
| I398 | Endocarditis, valve unspecified, in diseases classified elsewhere |
| I40 | Acute myocarditis |
| I400 | Infective myocarditis |
| I401 | Isolated myocarditis |
| I408 | Other acute myocarditis |
| I409 | Acute myocarditis, unspecified |
| I41 | Myocarditis in diseases classified elsewhere |
| I410 | Myocarditis in bacterial diseases classified elsewhere |
| I411 | Myocarditis in viral diseases classified elsewhere |
| I412 | Myocarditis in other infectious and parasitic diseases classified elsewhere |
| I418 | Myocarditis in other diseases classified elsewhere |
| I42 | Cardiomyopathy |
| I420 | Dilated cardiomyopathy |
| I421 | Obstructive hypertrophic cardiomyopathy |
| I422 | Other hypertrophic cardiomyopathy |
| I423 | Endomyocardial (eosinophilic) disease |
| I424 | Endocardial fibroelastosis |
| I425 | Other restrictive cardiomyopathy |
| I426 | Alcoholic cardiomyopathy |
| I427 | Cardiomyopathy due to drugs and other external agents |
| I428 | Other cardiomyopathies |
| I429 | Cardiomyopathy, unspecified |
| I43 | Cardiomyopathy in diseases classified elsewhere |
| I430 | Cardiomyopathy in infectious and parasitic diseases classified elsewhere |
| I431 | Cardiomyopathy in metabolic diseases |
| I432 | Cardiomyopathy in nutritional diseases |
| I438 | Cardiomyopathy in other diseases classified elsewhere |
| I44 | Atrioventricular and left bundle-branch block |
| I440 | Atrioventricular block, first degree |
| I441 | Atrioventricular block, second degree |
| I442 | Atrioventricular block, complete |
| I443 | Other and unspecified atrioventricular block |
| I444 | Left anterior fascicular block |
| I445 | Left posterior fascicular block |
| I446 | Other and unspecified fascicular block |
| I447 | Left bundle-branch block, unspecified |
| I45 | Other conduction disorders |
| I450 | Right fascicular block |
| I451 | Other and unspecified right bundle-branch block |
| I452 | Bifascicular block |
| I453 | Trifascicular block |
| I454 | Nonspecific intraventricular block |
| I455 | Other specified heart block |
| I456 | Pre-excitation syndrome |
| I458 | Other specified conduction disorders |
| I459 | Conduction disorder, unspecified |
| I46 | Cardiac arrest |
| I460 | Cardiac arrest with successful resuscitation |
| I461 | Sudden cardiac death, so described |
| I469 | Cardiac arrest, unspecified |
| I47 | Paroxysmal tachycardia |
| I470 | Re-entry ventricular arrhythmia |
| I471 | Supraventricular tachycardia |
| I472 | Ventricular tachycardia |
| I479 | Paroxysmal tachycardia, unspecified |
| I48 | Atrial fibrillation and flutter |
| I480 | Paroxysmal atrial fibrillation |
| I481 | Persistent atrial fibrillation |
| I482 | Chronic atrial fibrillation |
| I483 | Typical atrial flutter |
| I484 | Atypical atrial flutter |
| I489 | Atrial fibrillation and atrial flutter, unspecified |
| I49 | Other cardiac arrhythmias |
| I490 | Ventricular fibrillation and flutter |
| I491 | Atrial premature depolarization |
| I492 | Junctional premature depolarization |
| I493 | Ventricular premature depolarization |
| I494 | Other and unspecified premature depolarization |
| I495 | Sick sinus syndrome |
| I498 | Other specified cardiac arrhythmias |
| I499 | Cardiac arrhythmia, unspecified |
| I50 | Heart failure |
| I500 | Congestive heart failure |
| I501 | Left ventricular failure |
| I509 | Heart failure, unspecified |
| I51 | Complications and ill-defined descriptions of heart disease |
| I510 | Cardiac septal defect, acquired |
| I511 | Rupture of chordae tendineae, not elsewhere classified |
| I512 | Rupture of papillary muscle, not elsewhere classified |
| I513 | Intracardiac thrombosis, not elsewhere classified |
| I514 | Myocarditis, unspecified |
| I515 | Myocardial degeneration |
| I516 | Cardiovascular disease, unspecified |
| I517 | Cardiomegaly |
| I518 | Other ill-defined heart diseases |
| I519 | Heart disease, unspecified |
| I52 | Other heart disorders in diseases classified elsewhere |
| I520 | Other heart disorders in bacterial diseases classified elsewhere |
| I521 | Other heart disorders in other infectious and parasitic diseases classified elsewhere |
| I528 | Other heart disorders in other diseases classified elsewhere |
| I60 | Subarachnoid haemorrhage |
| I600 | Subarachnoid haemorrhage from carotid siphon and bifurcation |
| I601 | Subarachnoid haemorrhage from middle cerebral artery |
| I602 | Subarachnoid haemorrhage from anterior communicating artery |
| I603 | Subarachnoid haemorrhage from posterior communicating artery |
| I604 | Subarachnoid haemorrhage from basilar artery |
| I605 | Subarachnoid haemorrhage from vertebral artery |
| I606 | Subarachnoid haemorrhage from other intracranial arteries |
| I607 | Subarachnoid haemorrhage from intracranial artery, unspecified |
| I608 | Other subarachnoid haemorrhage |
| I609 | Subarachnoid haemorrhage, unspecified |
| I61 | Intracerebral haemorrhage |
| I610 | Intracerebral haemorrhage in hemisphere, subcortical |
| I611 | Intracerebral haemorrhage in hemisphere, cortical |
| I612 | Intracerebral haemorrhage in hemisphere, unspecified |
| I613 | Intracerebral haemorrhage in brain stem |
| I614 | Intracerebral haemorrhage in cerebellum |
| I615 | Intracerebral haemorrhage, intraventricular |
| I616 | Intracerebral haemorrhage, multiple localized |
| I618 | Other intracerebral haemorrhage |
| I619 | Intracerebral haemorrhage, unspecified |
| I62 | Other nontraumatic intracranial haemorrhage |
| I620 | Nontraumatic subdural haemorrhage |
| I621 | Nontraumatic extradural haemorrhage |
| I629 | Intracranial haemorrhage (nontraumatic), unspecified |
| I63 | Cerebral infarction |
| I630 | Cerebral infarction due to thrombosis of precerebral arteries |
| I631 | Cerebral infarction due to embolism of precerebral arteries |
| I632 | Cerebral infarction due to unspecified occlusion or stenosis of precerebral arteries |
| I633 | Cerebral infarction due to thrombosis of cerebral arteries |
| I634 | Cerebral infarction due to embolism of cerebral arteries |
| I635 | Cerebral infarction due to unspecified occlusion or stenosis of cerebral arteries |
| I636 | Cerebral infarction due to cerebral venous thrombosis, nonpyogenic |
| I638 | Other cerebral infarction |
| I639 | Cerebral infarction, unspecified |
| I64 | Stroke, not specified as haemorrhage or infarction |
| I65 | Occlusion and stenosis of precerebral arteries, not resulting in cerebral infarction |
| I650 | Occlusion and stenosis of vertebral artery |
| I651 | Occlusion and stenosis of basilar artery |
| I652 | Occlusion and stenosis of carotid artery |
| I653 | Occlusion and stenosis of multiple and bilateral precerebral arteries |
| I658 | Occlusion and stenosis of other precerebral artery |
| I659 | Occlusion and stenosis of unspecified precerebral artery |
| I66 | Occlusion and stenosis of cerebral arteries, not resulting in cerebral infarction |
| I660 | Occlusion and stenosis of middle cerebral artery |
| I661 | Occlusion and stenosis of anterior cerebral artery |
| I662 | Occlusion and stenosis of posterior cerebral artery |
| I663 | Occlusion and stenosis of cerebellar arteries |
| I664 | Occlusion and stenosis of multiple and bilateral cerebral arteries |
| I668 | Occlusion and stenosis of other cerebral artery |
| I669 | Occlusion and stenosis of unspecified cerebral artery |
| I67 | Other cerebrovascular diseases |
| I670 | Dissection of cerebral arteries, nonruptured |
| I671 | Cerebral aneurysm, nonruptured |
| I672 | Cerebral atherosclerosis |
| I673 | Progressive vascular leukoencephalopathy |
| I674 | Hypertensive encephalopathy |
| I675 | Moyamoya disease |
| I676 | Nonpyogenic thrombosis of intracranial venous system |
| I677 | Cerebral arteritis, not elsewhere classified |
| I678 | Other specified cerebrovascular diseases |
| I679 | Cerebrovascular disease, unspecified |
| I68 | Cerebrovascular disorders in diseases classified elsewhere |
| I680 | Cerebral amyloid angiopathy E85.- |
| I681 | Cerebral arteritis in infectious and parasitic diseases classified elsewhere |
| I682 | Cerebral arteritis in other diseases classified elsewhere |
| I688 | Other cerebrovascular disorders in diseases classified elsewhere |
| I69 | Sequelae of cerebrovascular disease |
| I690 | Sequelae of subarachnoid haemorrhage |
| I691 | Sequelae of intracerebral haemorrhage |
| I692 | Sequelae of other nontraumatic intracranial haemorrhage |
| I693 | Sequelae of cerebral infarction |
| I694 | Sequelae of stroke, not specified as haemorrhage or infarction |
| I698 | Sequelae of other and unspecified cerebrovascular diseases |
| I70 | Atherosclerosis |
| I700 | Atherosclerosis of aorta |
| I701 | Atherosclerosis of renal artery |
| I702 | Atherosclerosis of arteries of extremities |
| I708 | Atherosclerosis of other arteries |
| I709 | Generalized and unspecified atherosclerosis |
| I71 | Aortic aneurysm and dissection |
| I710 | Dissection of aorta [any part] |
| I711 | Thoracic aortic aneurysm, ruptured |
| I712 | Thoracic aortic aneurysm, without mention of rupture |
| I713 | Abdominal aortic aneurysm, ruptured |
| I714 | Abdominal aortic aneurysm, without mention of rupture |
| I715 | Thoracoabdominal aortic aneurysm, ruptured |
| I716 | Thoracoabdominal aortic aneurysm, without mention of rupture |
| I718 | Aortic aneurysm of unspecified site, ruptured |
| I719 | Aortic aneurysm of unspecified site, without mention of rupture |
| I72 | Other aneurysm and dissection |
| I720 | Aneurysm and dissection of carotid artery |
| I721 | Aneurysm and dissection of artery of upper extremity |
| I722 | Aneurysm and dissection of renal artery |
| I723 | Aneurysm and dissection of iliac artery |
| I724 | Aneurysm and dissection of artery of lower extremity |
| I725 | Aneurysm and dissection of other precerebral arteries |
| I726 | Aneurysm and dissection of vertebral artery |
| I728 | Aneurysm and dissection of other specified arteries |
| I729 | Aneurysm and dissection of unspecified site |
| I73 | Other peripheral vascular diseases |
| I730 | Raynaud syndrome |
| I731 | Thromboangiitis obliterans [Buerger] |
| I738 | Other specified peripheral vascular diseases |
| I739 | Peripheral vascular disease, unspecified |
| I74 | Arterial embolism and thrombosis |
| I740 | Embolism and thrombosis of abdominal aorta |
| I741 | Embolism and thrombosis of other and unspecified parts of aorta |
| I742 | Embolism and thrombosis of arteries of upper extremities |
| I743 | Embolism and thrombosis of arteries of lower extremities |
| I744 | Embolism and thrombosis of arteries of extremities, unspecified |
| I745 | Embolism and thrombosis of iliac artery |
| I748 | Embolism and thrombosis of other arteries |
| I749 | Embolism and thrombosis of unspecified artery |
| I77 | Other disorders of arteries and arterioles |
| I770 | Arteriovenous fistula, acquired |
| I771 | Stricture of artery |
| I772 | Rupture of artery |
| I773 | Arterial fibromuscular dysplasia |
| I774 | Coeliac artery compression syndrome |
| I775 | Necrosis of artery |
| I776 | Arteritis, unspecified |
| I778 | Other specified disorders of arteries and arterioles |
| I779 | Disorder of arteries and arterioles, unspecified |
| I78 | Diseases of capillaries |
| I780 | Hereditary haemorrhagic telangiectasia |
| I781 | Naevus, non-neoplastic |
| I788 | Other diseases of capillaries |
| I789 | Disease of capillaries, unspecified |
| I79 | Disorders of arteries, arterioles and capillaries in diseases classified elsewhere |
| I790 | Aneurysm of aorta in diseases classified elsewhere |
| I791 | Aortitis in diseases classified elsewhere |
| I792 | Peripheral angiopathy in diseases classified elsewhere |
| I798 | Other disorders of arteries, arterioles and capillaries in diseases classified elsewhere |
| I80 | Phlebitis and thrombophlebitis |
| I800 | Phlebitis and thrombophlebitis of superficial vessels of lower extremities |
| I801 | Phlebitis and thrombophlebitis of femoral vein |
| I802 | Phlebitis and thrombophlebitis of other deep vessels of lower extremities |
| I803 | Phlebitis and thrombophlebitis of lower extremities, unspecified |
| I808 | Phlebitis and thrombophlebitis of other sites |
| I809 | Phlebitis and thrombophlebitis of unspecified site |
| I81 | Portal vein thrombosis |
| I82 | Other venous embolism and thrombosis |
| I820 | Budd-Chiari syndrome |
| I821 | Thrombophlebitis migrans |
| I822 | Embolism and thrombosis of vena cava |
| I823 | Embolism and thrombosis of renal vein |
| I828 | Embolism and thrombosis of other specified veins |
| I829 | Embolism and thrombosis of unspecified vein |
| I83 | Varicose veins of lower extremities |
| I830 | Varicose veins of lower extremities with ulcer |
| I831 | Varicose veins of lower extremities with inflammation |
| I832 | Varicose veins of lower extremities with both ulcer and inflammation |
| I839 | Varicose veins of lower extremities without ulcer or inflammation |
| I85 | Oesophageal varices |
| I850 | Oesophageal varices with bleeding |
| I859 | Oesophageal varices without bleeding |
| I86 | Varicose veins of other sites |
| I860 | Sublingual varices |
| I861 | Scrotal varices |
| I862 | Pelvic varices |
| I863 | Vulval varices |
| I864 | Gastric varices |
| I868 | Varicose veins of other specified sites |
| I87 | Other disorders of veins |
| I870 | Postthrombotic syndrome |
| I871 | Compression of vein |
| I872 | Venous insufficiency (chronic)(peripheral) |
| I878 | Other specified disorders of veins |
| I879 | Disorder of vein, unspecified |
| I95 | Hypotension |
| I950 | Idiopathic hypotension |
| I951 | Orthostatic hypotension |
| I952 | Hypotension due to drugs |
| I958 | Other hypotension |
| I959 | Hypotension, unspecified |
| I97 | Postprocedural disorders of circulatory system, not elsewhere classified |
| I970 | Postcardiotomy syndrome |
| I971 | Other functional disturbances following cardiac surgery |
| I972 | Postmastectomy lymphoedema syndrome |
| I978 | Other postprocedural disorders of circulatory system, not elsewhere classified |
| I979 | Postprocedural disorder of circulatory system, unspecified |
| I98 | Other disorders of circulatory system in diseases classified elsewhere |
| I980 | Cardiovascular syphilis |
| I981 | Cardiovascular disorders in other infectious and parasitic diseases classified elsewhere |
| I982 | Oesophageal varices without bleeding in diseases classified elsewhere |
| I983 | Oesophageal varices with bleeding in diseases classified elsewhere |
| I988 | Other specified disorders of circulatory system in diseases classified elsewhere |
| I99 | Other and unspecified disorders of circulatory system |
| G450 | Vertebro-basilar artery syndrome |
| G451 | Carotid artery syndrome (hemispheric) |
| G452 | Multiple and bilateral precerebral artery syndromes |
| G453 | Amaurosis fugax |
| G454 | Transient global amnesia |
| G458 | Other transient cerebral ischaemic attacks and related syndromes |
| G459 | Transient cerebral ischaemic attack, unspecified |
| G460 | Middle cerebral artery syndrome |
| G461 | Anterior cerebral artery syndrome |
| G462 | Posterior cerebral artery syndrome |
| G463 | Brain stem stroke syndrome |
| G464 | Cerebellar stroke syndrome |
| G465 | Pure motor lacunar syndrome |
| G466 | Pure sensory lacunar syndrome |
| G467 | Other lacunar syndromes |
| G468 | Other vascular syndromes of brain in cerebrovascular diseases |
| H341 | Central retinal artery occlusion |
| O225 | Cerebral venous thrombosis in pregnancy |
| O873 | Cerebral venous thrombosis in the puerperium |
| O223 | Deep phlebothrombosis in pregnancy |
| O871 | Deep phlebothrombosis in the puerperium |
| O082 | Embolism following abortion and ectopic and molar pregnancy |
| O882 | Obstetric blood-clot embolism |

Supplementary Table S2 Full Baseline table

| **Characteristic** | **Overall**  N = 20,940 | **Rheumatoid Arthritis**  N = 17,669 | **Psoriatic Arthritis**  N = 3,271 |
| --- | --- | --- | --- |
| Age of diagnosis, Median (Q1, Q3); n / N (%) | 59 (47, 70) | 61 (49, 72) | 48 (36, 59) |
| 18-30 | 1,059 (5.1%) | 689 (3.9%) | 370 (11%) |
| 30-39 | 2,165 (10%) | 1,457 (8.2%) | 708 (22%) |
| 40-49 | 2,942 (14%) | 2,275 (13%) | 667 (20%) |
| 50-59 | 4,638 (22%) | 3,871 (22%) | 767 (23%) |
| 60-69 | 4,557 (22%) | 4,122 (23%) | 435 (13%) |
| 70-79 | 4,108 (20%) | 3,823 (22%) | 285 (8.7%) |
| 80+ | 1,471 (7.0%) | 1,432 (8.1%) | 39 (1.2%) |
| Gender, n / N (%) |  |  |  |
| Female | 13,004 (62%) | 11,197 (63%) | 1,807 (55%) |
| Male | 7,936 (38%) | 6,472 (37%) | 1,464 (45%) |
| IMD5, n / N (%) |  |  |  |
| 1 | 3,507 (17%) | 2,925 (17%) | 582 (18%) |
| 2 | 4,019 (19%) | 3,449 (20%) | 570 (17%) |
| 3 | 4,171 (20%) | 3,535 (20%) | 636 (19%) |
| 4 | 2,058 (9.8%) | 1,722 (9.7%) | 336 (10%) |
| 5 | 4,143 (20%) | 3,546 (20%) | 597 (18%) |
| Ethnicity, n / N (%) |  |  |  |
| White | 17,836 (85%) | 14,957 (85%) | 2,879 (88%) |
| Asian | 1,598 (7.6%) | 1,381 (7.8%) | 217 (6.6%) |
| Black | 498 (2.4%) | 471 (2.7%) | 27 (0.8%) |
| Other | 780 (3.7%) | 670 (3.8%) | 110 (3.4%) |
| Smoking status, n / N (%) |  |  |  |
| Never smoked | 9,068 (43%) | 7,539 (43%) | 1,529 (47%) |
| Current smoker | 3,928 (19%) | 3,374 (19%) | 554 (17%) |
| Ex-smoker | 5,955 (28%) | 5,124 (29%) | 831 (25%) |
| Patient in paid work, n / N (%) | 9,832 (47%) | 7,754 (44%) | 2,078 (64%) |
| Comorbidity, n / N (%) |  |  |  |
| Lung disease | 2,134 (10%) | 1,991 (11%) | 143 (4.4%) |
| Heart attack | 1,127 (5.4%) | 1,027 (5.8%) | 100 (3.1%) |
| Hypertension | 4,767 (23%) | 4,295 (24%) | 472 (14%) |
| Diabetes | 2,071 (9.9%) | 1,834 (10%) | 237 (7.2%) |
| Fracture | 425 (2.0%) | 381 (2.2%) | 44 (1.3%) |
| Cancer | 812 (3.9%) | 729 (4.1%) | 83 (2.5%) |
| Stomach ulcer | 708 (3.4%) | 628 (3.6%) | 80 (2.4%) |
| Depression | 1,609 (7.7%) | 1,292 (7.3%) | 317 (9.7%) |
| Seropositive, n / N (%) | 12,331 (59%) | 12,041 (68%) | 290 (8.9%) |
| Referral via EIA pathway, n / N (%) | 14,844 (71%) | 12,582 (71%) | 2,262 (69%) |
| Baseline corticosteroids, n / N (%) | 15,280 (73%) | 13,638 (77%) | 1,642 (50%) |
| DMARD treatment waiting time, Median (Q1, Q3) | 36 (20, 68) | 36 (20, 67) | 42 (22, 79) |
| Baseline DAS28, n / N (%) |  |  |  |
| Low | 2,900 (14%) | 2,224 (13%) | 676 (21%) |
| High | 8,534 (41%) | 7,767 (44%) | 767 (23%) |
| Moderate | 8,139 (39%) | 6,707 (38%) | 1,432 (44%) |
| EULAR Response (3-month), n / N (%) |  |  |  |
| No response | 4,478 (21%) | 3,756 (21%) | 722 (22%) |
| Good response | 4,798 (23%) | 4,345 (25%) | 453 (14%) |
| Moderate response | 3,980 (19%) | 3,509 (20%) | 471 (14%) |
| CCP, n / N (%) |  |  |  |
| Negative | 7,048 (34%) | 5,106 (29%) | 1,942 (59%) |
| Positive | 9,478 (45%) | 9,362 (53%) | 116 (3.5%) |
| CRP, Median (Q1, Q3); n / N (%) | 11 (4, 28) | 11 (4, 29) | 7 (3, 19) |
| Normal | 555 (2.7%) | 439 (2.5%) | 116 (3.5%) |
| Elevation | 18,233 (87%) | 15,543 (88%) | 2,690 (82%) |
| ESR, Median (Q1, Q3); n / N (%) | 26 (11, 44) | 27 (12, 45) | 16 (6, 33) |
| Normal | 6,086 (29%) | 4,963 (28%) | 1,123 (34%) |
| High | 7,018 (34%) | 6,199 (35%) | 819 (25%) |
| RDCI, n / N (%) |  |  |  |
| None | 11,574 (55%) | 9,429 (53%) | 2,145 (66%) |
| More than one | 3,326 (16%) | 3,015 (17%) | 311 (9.5%) |
| One | 5,847 (28%) | 5,071 (29%) | 776 (24%) |

Supplementary Figure S1 Crude incidence rate of interest outcome by age and gender with 95% confidence intervals estimated assuming a Poisson distribution in RA and PsA cohort

Supplementary Figure S2 Cumulative incidence of the outcome by ethnicity in RA

Supplementary Figure S3 Cumulative incidence of the outcome by ethnicity in PsA

Supplementary Figure S4 Cumulative incidence of the outcome by gender and ethnicity in PsA without positive RF

Supplementary Figure S5 Cumulative mortality by CVD and non CVD death in RA and PsA

Supplementary Table S3 Incidence in RA without HF history, PsA without HF history and PsA without RF positive.

|  | CVD  (n) | Proportion  (per 100 people)  95%CI | Incidence  (per 100 pys)  95%CI | MACE  (n) | Proportion  (per 100 people), 95%CI | Incidence  (per 100 pys)  95%CI | All-cause death  (n) | Proportion  (per 100 people)  95%CI | Incidence  (per 100 pys)  95%CI | CVD death  (n) | Proportion  (per 100 people)  95%CI | Incidence  (per 100 pys)  95%CI |
| --- | --- | --- | --- | --- | --- | --- | --- | --- | --- | --- | --- | --- |
| RA without HF history | 841 | 5.1 (4.77 – 5.45) | 1.91 (1.79 – 2.05) | 355 | 2.15 (1.94 – 2.39) | 0.81 (0.73 – 0.9) | 613 | 3.72 (3.44 – 4.02) | 1.4 (1.29 – 1.51) | 161 | 0.98 (0.83 – 1.14) | 0.37 (0.31 – 0.43) |
| Female | 453 | 4.24 (3.86 – 4.64) | 1.58 (1.44 – 1.73) | 181 | 1.69 (1.46 – 1.96) | 0.63 (0.54 – 0.73) | 314 | 2.94 (2.63 – 3.27) | 1.1 (0.98 – 1.22) | 72 | 0.67 (0.53 – 0.85) | 0.25 (0.2 – 0.32) |
| Male | 388 | 6.71 (3.86 – 4.64) | 2.54 (1.44 – 1.73) | 174 | 3.01 (1.46 – 1.96) | 1.14 (0.54 – 0.73) | 299 | 5.17 (2.63 – 3.27) | 1.96 (0.98 – 1.22) | 89 | 1.54 (0.53 – 0.85) | 0.58 (0.2 – 0.32) |
| PsA without HF history | 87 | 2.78 (2.23 – 3.42) | 1.04 (0.83 – 1.28) | 18 | 0.57 (0.34 – 0.91) | 0.22 (0.13 – 0.34) | 32 | 1.02 (0.7 – 1.44) | 0.38 (0.26 – 0.54) | 5 | 0.16 (0.05 – 0.37) | 0.06 (0.02 – 0.14) |
| Female | 36 | 2.07 (1.45 – 2.86) | 0.77 (0.54 – 1.07) | 6 | 0.35 (0.13 – 0.75) | 0.13 (0.05 – 0.28) | 15 | 0.86 (0.48 – 1.42) | 0.32 (0.18 – 0.53) | 2 | 0.12 (0.01 – 0.42) | 0.04 (0.01 – 0.15) |
| Male | 51 | 3.66 (1.45 – 2.86) | 1.38 (0.54 – 1.07) | 12 | 0.86 (0.13 – 0.75) | 0.33 (0.05 – 0.28) | 17 | 1.22 (0.48 – 1.42) | 0.46 (0.18 – 0.53) | 3 | 0.22 (0.01 – 0.42) | 0.08 (0.01 – 0.15) |
| PsA without RF positive | 75 | 3.2 (2.53 – 4) | 1.21 (0.95 – 1.51) | 15 | 0.64 (0.36 – 1.05) | 0.24 (0.14 – 0.4) | 21 | 0.9 (0.56 – 1.37) | 0.34 (0.21 – 0.52) | 4 | 0.17 (0.05 – 0.44) | 0.06 (0.02 – 0.16) |
| Female | 27 | 2.11 (1.4 – 3.06) | 0.8 (0.53 – 1.16) | 5 | 0.39 (0.13 – 0.91) | 0.15 (0.05 – 0.35) | 11 | 0.86 (0.43 – 1.53) | 0.33 (0.16 – 0.58) | 2 | 0.16 (0.02 – 0.56) | 0.06 (0.01 – 0.21) |
| Male | 48 | 4.51 (1.4 – 3.06) | 1.69 (0.53 – 1.16) | 10 | 0.94 (0.13 – 0.91) | 0.35 (0.05 – 0.35) | 10 | 0.94 (0.43 – 1.53) | 0.35 (0.16 – 0.58) | 2 | 0.19 (0.02 – 0.56) | 0.07 (0.01 – 0.21)  > |

Supplementary Table S4 Standardized incidence ratios and Standardized mortality ratios of RA patients compared with the general population

| Group | CVD (n) | SIR 95%CI | Death (n) | SMR 95%CI |
| --- | --- | --- | --- | --- |
| Overall | 1012 | 1.25, (1.18 - 1.33) | 744 | 1.11, (1.03 - 1.19) |
| Female |  |  |  |  |
| 18-40 | 7 | 1.73, (0.69 - 3.57) | 2 | 1.03, (0.12 - 3.73) |
| 40-49 | 32 | 2.09, (1.43 - 2.95) | 8 | 1.39, (0.6 - 2.74) |
| 50-59 | 74 | 1.39, (1.09 - 1.75) | 31 | 1.53, (1.04 - 2.17) |
| 60-69 | 105 | 0.88, (0.72 - 1.06) | 63 | 1.48, (1.14 - 1.89) |
| 70-79 | 189 | 1.13, (0.98 - 1.31) | 127 | 1.24, (1.03 - 1.48) |
| 80+ | 107 | 1.42, (1.17 - 1.72) | 130 | 1.04, (0.87 - 1.23) |
| Overall | 514 | 1.18, (1.08 - 1.29) | 361 | 1.21, (1.09 - 1.34) |
| Under 65 | 166 | 1.32, (1.13 - 1.54) | 70 | 1.52, (1.18 - 1.92) |
| Over 65 | 348 | 1.13, (1.01 - 1.25) | 291 | 1.15, (1.03 - 1.3) |
| Under 80 | 407 | 1.13, (1.03 - 1.25) | 231 | 1.33, (1.17 - 1.52) |
| Male |  |  |  |  |
| 18-40 | 8 | 5.46, (2.35 - 10.76) | 2 | 1.97, (0.22 - 7.11) |
| 40-49 | 13 | 1.33, (0.71 - 2.28) | 7 | 1.83, (0.73 - 3.77) |
| 50-59 | 62 | 1.16, (0.89 - 1.49) | 32 | 1.88, (1.28 - 2.65) |
| 60-69 | 139 | 1.21, (1.02 - 1.43) | 71 | 1.45, (1.13 - 1.82) |
| 70-79 | 168 | 1.21, (1.03 - 1.41) | 156 | 1.37, (1.16 - 1.60) |
| 80+ | 108 | 1.99, (1.63 - 2.4) | 115 | 1.00, (0.82 - 1.19) |
| Overall | 498 | 1.34, (1.22 – 1.46) | 383 | 1.27, (1.15 - 1.41) |
| Under 65 | 147 | 1.31, (1.11 - 1.54) | 70 | 1.81, (1.41 - 2.28) |
| Over 65 | 351 | 1.35, (1.21 - 1.49) | 313 | 1.19, (1.07 - 1.33) |
| Under 80 | 390 | 1.22, (1.10 – 1.35) | 268 | 1.45, (1.28 - 1.63) |

Supplementary Table S5 Standardized incidence ratios and Standardized mortality ratios of PsA patients compared with the general population

| Group | CVD (n) | SIR_95%CI | Death (n) | SMR_95%CI |
| --- | --- | --- | --- | --- |
| Overall | 104 | 1.26 (1.03 - 1.53) | 38 | 0.88 (0.62 - 1.21) |
| Female |  |  |  |  |
| 18-40 | 7 | 4.9, (1.96 - 10.1) | 0 | - |
| 40-49 | 6 | 1.83, (0.67 - 3.98) | 4 | 3.31, (0.89 - 8.47) |
| 50-59 | 13 | 1.36, (0.73 - 2.33) | 2 | 0.55, (0.06 - 1.98) |
| 60-69 | 8 | 0.75, (0.32 - 1.48) | 3 | 0.79, (0.16 - 2.31) |
| 70-79 | 4 | 0.33, (0.09 - 0.85) | 8 | 1.2, (0.52 - 2.36) |
| 80+ | 3 | 1.37, (0.28 - 4.01) | 2 | 0.54, (0.06 - 1.95) |
| Overall | 41 | 1.02, (0.74 - 1.39) | 19 | 2.71, (1.63 - 4.24) |
| Under 65 | 29 | 1.52, (1.02 - 2.19) | 8 | 1.12, (0.48 - 2.2) |
| Over 65 | 12 | 0.6, (0.31 - 1.04) | 11 | 0.88, (0.44 - 1.57) |
| Under 80 | 38 | 1.03, (0.73 - 1.41) | 15 | 2.5, (1.40 - 4.12) |
| Male |  |  |  |  |
| 18-40 | 7 | 4.47, (1.79 - 9.2) | 1 | 1.0, (0.01 - 5.14) |
| 40-49 | 11 | 2.61, (1.3 - 4.66) | 2 | 1.19, (0.13 - 4.28) |
| 50-59 | 11 | 1.0, (0.47 - 1.67) | 2 | 0.54, (0.06 - 1.94) |
| 60-69 | 14 | 1.05, (0.57 - 1.76) | 6 | 1.1, (0.4 - 2.39) |
| 70-79 | 16 | 1.51, (0.86 - 2.44) | 7 | 0.82, (0.33 - 1.68) |
| 80+ | 4 | 2.19, (0.59 - 5.6) | 1 | 0.28, (0 - 1.55) |
| Overall | 63 | 1.46, (1.13 - 1.87) | 19 | 0.80, (0.48 - 1.24) |
| Under 65 | 40 | 1.59, (1.13 - 2.16) | 6 | 0.65, (0.24 - 1.41) |
| Over 65 | 23 | 1.27, (0.8 - 1.9) | 13 | 0.88, (0.47 - 1.5) |
| Under 80 | 59 | 1.40, (1.07 - 1.81) | 18 | 1.0, (0.53 - 1.42) |

Supplementary Table S6 Regression results in RA

|  | RA cohort |  |  |  | RA no HF history |  |  |  | RA no cancer |  |
| --- | --- | --- | --- | --- | --- | --- | --- | --- | --- | --- |
|  | Any CVD | MACE | Death | CVD death | Any CVD | MACE | Death | CVD death | Death | CVD death |
| IMD5 |  |  |  |  |  |  |  |  |  |  |
| 1 (ref) |  |  |  |  |  |  |  |  |  |  |
| 2 | 0.94 (0.77-1.15, p=0.530) | 0.86 (0.63-1.17, p=0.330) | 0.97 (0.77-1.23, p=0.822) | 0.66 (0.42-1.07, p=0.090) | 0.97 (0.78-1.20, p=0.780) | 0.98 (0.69-1.38, p=0.900) | 1.04 (0.80-1.35, p=0.775) | 0.77 (0.47-1.28, p=0.320) | 1.00 (0.78-1.28, p=0.999) | 0.64 (0.39-1.05, p=0.079) |
| 3 | 1.12 (0.92-1.36, p=0.250) | 1.04 (0.77-1.40, p=0.820) | 1.05 (0.83-1.32, p=0.710) | 0.91 (0.59-1.40, p=0.670) | 1.08 (0.88-1.34, p=0.460) | 1.08 (0.77-1.51, p=0.670) | 1.11 (0.86-1.43, p=0.432) | 0.89 (0.55-1.45, p=0.640) | 1.08 (0.85-1.38, p=0.521) | 0.96 (0.62-1.50, p=0.860) |
| 4 | 1.00 (0.78-1.28, p=0.990) | 1.13 (0.79-1.62, p=0.510) | 1.04 (0.78-1.40, p=0.776) | 1.10 (0.66-1.84, p=0.710) | 0.96 (0.73-1.26, p=0.750) | 1.29 (0.87-1.92, p=0.210) | 1.15 (0.83-1.58, p=0.404) | 1.10 (0.61-1.96, p=0.760) | 1.05 (0.77-1.43, p=0.767) | 1.09 (0.64-1.87, p=0.750) |
| 5 | 0.95 (0.77-1.16, p=0.590) | 1.08 (0.79-1.46, p=0.630) | 1.22 (0.97-1.54, p=0.092) | 1.09 (0.71-1.68, p=0.690) | 0.96 (0.77-1.20, p=0.710) | 1.15 (0.81-1.62, p=0.430) | 1.32 (1.02-1.71, p=0.034) | 1.15 (0.71-1.85, p=0.580) | 1.19 (0.93-1.52, p=0.176) | 1.03 (0.65-1.61, p=0.910) |
| Ethnicity |  |  |  |  |  |  |  |  |  |  |
| White (ref) |  |  |  |  |  |  |  |  |  |  |
| Asian | 0.90 (0.65-1.24, p=0.520) | 0.51 (0.26-0.99, p=0.046) | 0.59 (0.35-0.99, p=0.046) | 0.55 (0.20-1.49, p=0.240) | 0.77 (0.53-1.11, p=0.160) | 0.40 (0.17-0.90, p=0.026) | 0.51 (0.28-0.92, p=0.027) | 0.32 (0.08-1.32, p=0.120) | 0.62 (0.37-1.04, p=0.069) | 0.56 (0.21-1.51, p=0.250) |
| Black | 0.94 (0.59-1.50, p=0.790) | 0.37 (0.12-1.17, p=0.091) | 0.79 (0.42-1.47, p=0.456) | 0.30 (0.04-2.14, p=0.230) | 0.98 (0.60-1.61, p=0.940) | 0.45 (0.14-1.40, p=0.170) | 0.76 (0.38-1.53, p=0.442) | 0.36 (0.05-2.55, p=0.310) | 0.92 (0.49-1.71, p=0.782) | 0.34 (0.05-2.39, p=0.280) |
| Other | 0.85 (0.55-1.30, p=0.440) | 0.47 (0.20-1.15, p=0.097) | 0.42 (0.20-0.89, p=0.023) | 0.45 (0.11-1.84, p=0.270) | 0.85 (0.54-1.34, p=0.480) | 0.56 (0.23-1.36, p=0.200) | 0.43 (0.19-0.97, p=0.042) | 0.54 (0.13-2.18, p=0.380) | 0.36 (0.15-0.88, p=0.025) | 0.26 (0.04-1.87, p=0.180) |
| Smoking status |  |  |  |  |  |  |  |  |  |  |
| Never smkoing (ref) |  |  |  |  |  |  |  |  |  |  |
| Current smoker | 1.67 (1.40-1.99, p<0.001) | 2.81 (2.17-3.65, p<0.001) | 3.11 (2.52-3.85, p<0.001) | 3.08 (2.10-4.50, p<0.001) | 1.75 (1.45-2.12, p<0.001) | 2.64 (1.99-3.51, p<0.001) | 2.95 (2.34-3.72, p<0.001) | 2.91 (1.94-4.38, p<0.001) | 3.05 (2.44-3.80, p<0.001) | 2.72 (1.83-4.02, p<0.001) |
| Ex-smoker | 1.29 (1.11-1.50, p=0.001) | 1.36 (1.06-1.74, p=0.014) | 1.63 (1.36-1.95, p<0.001) | 1.26 (0.88-1.80, p=0.210) | 1.30 (1.10-1.53, p=0.002) | 1.40 (1.07-1.82, p=0.014) | 1.64 (1.34-1.99, p<0.001) | 1.33 (0.90-1.96, p=0.150) | 1.64 (1.35-1.98, p<0.001) | 1.25 (0.87-1.81, p=0.230) |
| Comorbidity |  |  |  |  |  |  |  |  |  |  |
| Lung disease | 1.49 (1.28-1.75, p<0.001) | 1.60 (1.27-2.01, p<0.001) | 2.26 (1.92-2.65, p<0.001) | 2.14 (1.58-2.91, p<0.001) | 1.53 (1.29-1.82, p<0.001) | 1.63 (1.26-2.11, p<0.001) | 2.24 (1.87-2.67, p<0.001) | 2.19 (1.55-3.09, p<0.001) | 2.18 (1.83-2.58, p<0.001) | 1.90 (1.37-2.64, p<0.001) |
| Heart attack | 1.94 (1.62-2.31, p<0.001) | 1.80 (1.38-2.34, p<0.001) | 1.73 (1.42-2.10, p<0.001) | 1.76 (1.22-2.54, p=0.002) | - | - | - | - | 1.73 (1.40-2.13, p<0.001) | 1.65 (1.11-2.44, p=0.012) |
| Fracture | 1.27 (0.90-1.79, p=0.170) | 1.11 (0.63-1.94, p=0.720) | 1.20 (0.82-1.77, p=0.348) | 1.43 (0.69-2.95, p=0.330) | 1.16 (0.79-1.72, p=0.450) | 1.13 (0.61-2.07, p=0.700) | 1.37 (0.91-2.04, p=0.127) | 1.53 (0.70-3.32, p=0.280) | 1.30 (0.86-1.96, p=0.207) | 1.75 (0.85-3.60, p=0.130) |
| Cancer | 1.09 (0.85-1.40, p=0.510) | 0.91 (0.61-1.36, p=0.660) | 1.42 (1.12-1.81, p=0.004) | 1.07 (0.63-1.80, p=0.800) | 1.07 (0.81-1.42, p=0.620) | 0.79 (0.49-1.27, p=0.340) | 1.44 (1.10-1.89, p=0.008) | 0.94 (0.50-1.75, p=0.840) |  | - |
| Stomach ulcer | 1.05 (0.80-1.39, p=0.720) | 1.12 (0.74-1.69, p=0.590) | 1.11 (0.82-1.50, p=0.509) | 1.37 (0.79-2.37, p=0.260) | 1.15 (0.86-1.55, p=0.340) | 1.16 (0.74-1.82, p=0.530) | 1.11 (0.80-1.56, p=0.528) | 1.22 (0.64-2.33, p=0.550) | 1.11 (0.80-1.54, p=0.538) | 1.32 (0.73-2.38, p=0.350) |
| Depression | 1.13 (0.87-1.47, p=0.340) | 1.44 (1.00-2.09, p=0.051) | 1.70 (1.29-2.25, p<0.001) | 1.79 (1.07-3.01, p=0.027) | 1.20 (0.91-1.58, p=0.200) | 1.49 (1.00-2.21, p=0.050) | 1.75 (1.30-2.37, p<0.001) | 1.86 (1.07-3.26, p=0.029) | 1.81 (1.35-2.41, p<0.001) | 1.97 (1.17-3.32, p=0.011) |
| Hypertension_all records | 1.51 (1.32-1.73, p<0.001) | 1.43 (1.17-1.74, p=0.001) | 1.17 (1.01-1.36, p=0.042) | 1.38 (1.03-1.85, p=0.029) | 1.48 (1.28-1.72, p<0.001) | 1.41 (1.13-1.76, p=0.002) | 1.18 (1.00-1.39, p=0.051) | 1.50 (1.09-2.07, p=0.013) | 1.23 (1.05-1.45, p=0.009) | 1.41 (1.04-1.91, p=0.028) |
| Hypertension_baseline | 1.30 (1.14-1.49, p<0.001) | 1.24 (1.01-1.52, p=0.042) | 1.03 (0.88-1.20, p=0.711) | 1.21 (0.90-1.63, p=0.200) | 1.32 (1.13-1.53, p<0.001) | 1.21 (0.96-1.51, p=0.100) | 1.04 (0.87-1.23, p=0.678) | 1.27 (0.92-1.77, p=0.150) | 1.73 (1.40-2.13, p<0.001) | 1.23 (0.90-1.67, p=0.200) |
| Diabetes_all records | 1.44 (1.22-1.70, p<0.001) | 1.64 (1.29-2.08, p<0.001) | 1.59 (1.33-1.91, p<0.001) | 1.91 (1.38-2.66, p<0.001) | 1.42 (1.18-1.71, p<0.001) | 1.69 (1.30-2.21, p<0.001) | 1.53 (1.25-1.88, p<0.001) | 2.04 (1.41-2.96, p<0.001) | 1.63 (1.35-1.97, p<0.001) | 1.94 (1.37-2.74, p<0.001) |
| Diabetes_baseline | 1.42 (1.20-1.68, p<0.001) | 1.57 (1.22-2.01, p<0.001) | 1.51 (1.25-1.82, p<0.001) | 1.75 (1.23-2.48, p=0.002) | 1.38 (1.13-1.67, p=0.001) | 1.58 (1.19-2.09, p=0.001) | 1.42 (1.15-1.76, p=0.001) | 1.82 (1.23-2.70, p=0.003) | 1.59 (1.31-1.93, p<0.001) | 1.83 (1.28-2.62, p=0.001) |
| DAS28 Baselibne |  |  |  |  |  |  |  |  |  |  |
| Low (ref) |  |  |  |  |  |  |  |  |  |  |
| High_complete case | 1.11 (0.90-1.36, p=0.330) | 1.21 (0.87-1.68, p=0.250) | 1.39 (1.07-1.80, p=0.014) | 1.26 (0.79-2.01, p=0.330) | 1.16 (0.93-1.46, p=0.190) | 1.25 (0.86-1.80, p=0.240) | 1.43 (1.07-1.91, p=0.016) | 1.48 (0.86-2.55, p=0.160) | 1.45 (1.10-1.92, p=0.009) | 1.22 (0.75-1.97, p=0.420) |
| Moderate_complete case | 0.92 (0.74-1.14, p=0.450) | 1.00 (0.70-1.39, p=0.940) | 1.10 (0.83-1.45, p=0.511) | 0.77 (0.47-1.29, p=0.320) | 1.00 (0.78-1.25, p=0.930) | 1.09 (0.74-1.60, p=0.660) | 1.10 (0.81-1.49, p=0.558) | 0.88 (0.49-1.59, p=0.670) | 1.18 (0.88-1.58, p=0.282) | 0.76 (0.45-1.28, p=0.310) |
| High_MI | 1.13 (0.92 - 1.38, p=0.256) | 1.22 (0.88 - 1.69, p=0.225) | 1.39 (1.07 - 1.8, p=0.012) | 1.26 (0.79 - 2.01, p=0.323) | 1.19 (0.95 - 1.49, p=0.134) | 1.26 (0.87 - 1.81, p=0.222) | 1.37 (1.02 - 1.83, p=0.036) | 1.46 (0.83 - 2.57, p=0.194) | 1.43 (1.08 - 1.89, p=0.013) | 1.23 (0.76 - 1.98, p=0.395) |
| Moderate_MI | 0.95 (0.77 - 1.18, p=0.66) | 1 (0.71 - 1.42, p=0.98) | 1.11 (0.84 - 1.45, p=0.46) | 0.89 (0.49 - 1.31, p=0.372) | 1.00 (0.79 - 1.26, p=0.984) | 1.1 (0.75 - 1.62, p=0.619) | 1.06 (0.79 - 1.44, p=0.688) | 0.91 (0.48 - 1.7, p=0.757) | 1.17 (0.88 - 1.56, p=0.291) | 0.77 (0.46 - 1.29, p=0.323) |
| Seropositive | 1.07 (0.94-1.24, p=0.310) | 1.41 (1.13-1.76, p=0.002) | 1.80 (1.51-2.14, p<0.001) | 1.89 (1.35-2.65, p<0.001) | 1.06 (0.91-1.23, p=0.450) | 1.33 (1.04-1.69, p=0.020) | 1.79 (1.48-2.16, p<0.001) | 1.80 (1.24-2.61, p=0.002) | 1.80 (1.50-2.15, p<0.001) | 1.92 (1.34-2.74, p<0.001) |
| Elevation CRP baseline_complete case | 1.23 (0.72-2.09, p=0.450) | 1.66 (0.62-4.47, p=0.320) | 1.84 (0.82-4.11, p=0.137) | 1.47 (0.36-5.97, p=0.590) | 1.03 (0.61-1.76, p=0.900) | 1.38 (0.51-3.71, p=0.530) | 2.31 (0.86-6.18, p=0.095) | 1.21 (0.30-4.92, p=0.790) | 2.09 (0.87-5.04, p=0.101) | 1.43 (0.35-5.80, p=0.620) |
| Elevation CRP baseline_MI | 1.19 (0.7 - 2.01, p=0.518) | 1.61 (0.59 - 4.37, p=0.352) | 1.67 (0.67 - 4.14, p=0.274) | 1.34 (0.35 - 5.07, p=0.672) | 1.06 (0.62 - 1.82, p=0.82) | 1.37 (0.51 - 3.67, p=0.536) | 1.7 (0.72 - 4.03, p=0.229) | 1.00 (0.23 - 4.1, p=0.975) | 2.01 (0.77 - 5.22, p=0.157) | 1.5 (0.37 - 6.08, p=0.571) |
| High ESR baseline_complete case | 1.05 (0.89-1.22, p=0.580) | 1.06 (0.83-1.36, p=0.630) | 1.46 (1.20-1.77, p<0.001) | 1.45 (0.99-2.13, p=0.059) | 1.06 (0.90-1.26, p=0.480) | 1.03 (0.79-1.36, p=0.810) | 1.45 (1.17-1.79, p=0.001) | 1.42 (0.93-2.18, p=0.100) | 1.42 (1.16-1.75, p=0.001) | 1.54 (1.03-2.30, p=0.036) |
| High ESR baseline_MI | 1.01 (0.88 - 1.15, p=0.934) | 1.06 (0.85 - 1.33, p=0.616) | 1.39 (1.17 - 1.65, p<0.001) | 1.31 (0.94 - 1.81, p=0.11) | 1.01 (0.86 - 1.18, p=0.922) | 1.05 (0.82 - 1.33, p=0.712) | 1.37 (1.12 - 1.67, p=0.003) | 1.42 (0.93 - 2.17, p=0.109) | 1.36 (1.13 - 1.65, p=0.002) | 1.39 (0.97 - 1.99, p=0.075) |
| CCP Positive | 1.08 (0.94-1.25, p=0.280) | 1.55 (1.24-1.95, p<0.001) | 1.75 (1.47-2.08, p<0.001) | 2.09 (1.48-2.95, p<0.001) | 1.05 (0.90-1.23, p=0.530) | 1.49 (1.16-1.91, p=0.002) | 1.79 (1.48-2.17, p<0.001) | 2.00 (1.36-2.92, p<0.001) | 1.71 (1.43-2.05, p<0.001) | 2.03 (1.42-2.92, p<0.001) |
| Start corticosteroids | 1.00 (0.85-1.17, p=0.960) | 0.98 (0.76-1.25, p=0.840) | 0.95 (0.78-1.14, p=0.562) | 1.10 (0.75-1.61, p=0.610) | 0.96 (0.81-1.14, p=0.660) | 0.88 (0.68-1.15, p=0.360) | 0.92 (0.75-1.13, p=0.425) | 1.04 (0.69-1.57, p=0.840) | 0.93 (0.76-1.13, p=0.481) | 1.15 (0.78-1.72, p=0.480) |
| Eular Response 3 month |  |  |  |  |  |  |  |  |  |  |
| No response (ref) |  |  |  |  |  |  |  |  |  |  |
| Good response_complete case | 0.83 (0.68-0.99, p=0.044) | 0.72 (0.54-0.96, p=0.027) | 0.61 (0.48-0.77, p<0.001) | 0.62 (0.41-0.96, p=0.031) | 0.83 (0.68-1.02, p=0.079) | 0.68 (0.49-0.94, p=0.019) | 0.54 (0.42-0.70, p<0.001) | 0.57 (0.35-0.92, p=0.022) | 0.63 (0.49-0.80, p<0.001) | 0.60 (0.39-0.93, p=0.023) |
| Moderate response_complete case | 0.99 (0.82-1.20, p=0.910) | 0.90 (0.67-1.21, p=0.510) | 0.82 (0.65-1.03, p=0.093) | 0.76 (0.49-1.19, p=0.230) | 0.97 (0.79-1.19, p=0.770) | 0.90 (0.65-1.24, p=0.520) | 0.79 (0.61-1.01, p=0.061) | 0.79 (0.48-1.30, p=0.350) | 0.83 (0.65-1.06, p=0.131) | 0.70 (0.44-1.11, p=0.130) |
| Good response_MI | 0.80 (0.69 - 0.93, p=0.004) | 0.7 (0.55 - 0.88, p=0.003) | 0.53 (0.44 - 0.64, p<0.001) | 0.58 (0.41 - 0.82, p=0.002) | 0.79 (0.67 - 0.93, p=0.005) | 0.64 (0.49 - 0.84, p=0.001) | 0.47 (0.38 - 0.59, p<0.001) | 0.49 (0.32 - 0.73, p<0.001) | 0.54 (0.44 - 0.66, p<0.001) | 0.57 (0.39 - 0.83, p=0.003) |
| Moderate response_MI | 0.94 (0.8 - 1.1, p=0.45) | 0.85 (0.66 - 1.09, p=0.195) | 0.75 (0.62 - 0.9, p=0.003) | 0.71 (0.49 - 1.03, p=0.069) | 0.91 (0.77 - 1.09, p=0.308 | 0.84 (0.64 - 1.09, p=0.19) | 0.72 (0.58 - 0.88, p=0.002) | 0.66 (0.44 - 1.01, p=0.059) | 0.73 (0.6 - 0.89, p=0.003) | 0.66 (0.43 - 1, p=0.054) |

Supplementary Table S7 Regression result in PsA

|  | PsA cohort |  |  |  | PsA without RF positive |  |  |  | PsA without HF history |  |  |  |
| --- | --- | --- | --- | --- | --- | --- | --- | --- | --- | --- | --- | --- |
|  | Any CVD | MACE | Death | CVD death | Any CVD | MACE | Death | CVD death | Any CVD | MACE | Death | CVD death |
| IMD5 |  |  |  |  |  |  |  |  |  |  |  |  |
| 1 (ref) |  |  |  |  |  |  |  |  |  |  |  |  |
| 2 | 1.14 (0.62-2.12, p=0.670) | 0.71 (0.16-3.22, p=0.660) | 0.58 (0.14-2.45, p=0.462) | 0.98 (0.07-14.48, p=0.990) | 1.35 (0.62-2.95, p=0.450) | 0.99 (0.14-7.00, p=1.000) | 0.69 (0.15-3.12, p=0.632) | 0.31 (0.05-2.08, p=0.230) | 1.25 (0.64-2.46, p=0.510) | 0.50 (0.09-2.68, p=0.420) | 0.50 (0.09-2.71, p=0.418) | 0.35 (0.03-3.66, p=0.380) |
| 3 | 1.19 (0.65-2.19, p=0.580) | 1.54 (0.43-5.53, p=0.510) | 1.42 (0.45-4.47, p=0.553) | 1.04 (0.06-17.02, p=0.980) | 1.75 (0.83-3.71, p=0.140) | 2.29 (0.40-12.91, p=0.350) | 0.27 (0.03-2.39, p=0.237) | - | 1.34 (0.69-2.60, p=0.390) | 1.58 (0.44-5.61, p=0.480) | 1.26 (0.34-4.71, p=0.729) | 1.38 (0.28-6.79, p=0.695) |
| 4 | 0.92 (0.42-2.02, p=0.840) | 1.71 (0.38-7.75, p=0.490) | 2.15 (0.62-7.46, p=0.228) | - | 0.93 (0.32-2.68, p=0.890) | 1.37 (0.11-17.22, p=0.810) | 2.32 (0.57-9.46, p=0.241) | 0.98 (0.16-6.00, p=0.983) | 1.13 (0.50-2.57, p=0.760) | 1.10 (0.20-6.01, p=0.920) | 2.07 (0.52-8.31, p=0.305) | 1.33 (0.24-7.29, p=0.742) |
| 5 | 0.89 (0.45-1.74, p=0.730) | 0.65 (0.12-3.58, p=0.620) | 2.30 (0.77-6.89, p=0.137) | 1.27 (0.08-20.13, p=0.860) | 1.21 (0.55-2.70, p=0.640) | 1.35 (0.19-9.70, p=0.760) | 1.69 (0.44-6.44, p=0.444) | 0.67 (0.12-3.79, p=0.650) | 0.86 (0.40-1.81, p=0.680) | 0.32 (0.04-2.86, p=0.310) | 2.55 (0.76-8.52, p=0.128) | 0.77 (0.19-3.21, p=0.724) |
| Ethnicity |  |  |  |  |  |  |  |  |  |  |  |  |
| White (ref) |  |  |  |  |  |  |  |  |  |  |  |  |
| Asian | 0.80 (0.29-2.18, p=0.660) | - | 0.86 (0.12-6.36, p=0.885) | - | 1.03 (0.37-2.85, p=0.950) | - | 1.51 (0.20-11.47, p=0.692) |  | 0.91 (0.33-2.49, p=0.860) | - | 0.96 (0.13-7.10, p=0.967) | 3.85 (0.43-34.40, p=0.227) |
| Black | - | - | - | - | - | - | - |  | - | - | - | - |
| Other | 1.08 (0.27-4.38, p=0.920) | - | 2.73 (0.37-20.37, p=0.327) | - | 1.73 (0.42-7.09, p=0.440) | - | - |  | - | - | - | - |
| Smoking status |  |  |  |  |  |  |  |  |  |  |  |  |
| Never smkoing (ref) |  |  |  |  |  |  |  |  |  |  |  |  |
| Current smoker | 3.62 (2.14-6.13, p<0.001) | 1.51 (0.39-5.84, p=0.550) | 2.54 (1.04-6.19, p=0.041) | 4.07 (0.23-70.81, p=0.340) | 3.15 (1.68-5.90, p<0.001) | 0.90 (0.10-7.89, p=0.920) | 1.89 (0.49-7.24, p=0.351) | 2.04 (0.33-12.47, p=0.442) | 3.63 (2.05-6.40, p<0.001) | 1.17 (0.24-5.65, p=0.850) | 3.34 (1.30-8.55, p=0.012) | 1.43 (0.48-4.22, p=0.522) |
| Ex-smoker | 2.37 (1.44-3.91, p=0.001) | 1.44 (0.55-3.78, p=0.460) | 0.91 (0.40-2.08, p=0.831) | 1.02 (0.07-15.78, p=0.990) | 2.10 (1.18-3.72, p=0.011) | 1.47 (0.44-4.88, p=0.530) | 0.94 (0.34-2.62, p=0.907) | 1.55 (0.47-5.09, p=0.467) | 2.50 (1.45-4.32, p=0.001) | 1.08 (0.35-3.35, p=0.890) | 0.85 (0.33-2.19, p=0.735) | 1.45 (0.50-4.24, p=0.498) |
| Comorbidity |  |  |  |  |  |  |  |  |  |  |  |  |
| Lung disease | 1.09 (0.48-2.51, p=0.830) | 1.04 (0.23-4.74, p=0.960) | 1.02 (0.31-3.37, p=0.979) | 3.56 (0.36-35.00, p=0.280) | 1.25 (0.51-3.07, p=0.630) | 0.66 (0.09-5.08, p=0.690) | 1.68 (0.48-5.88, p=0.419) | 0.57 (0.12-2.58, p=0.463) | 1.33 (0.54-3.28, p=0.530) | 1.84 (0.41-8.34, p=0.430) | 1.47 (0.44-4.92, p=0.531) | 0.52 (0.12-2.23, p=0.379) |
| Heart attack | 2.85 (1.56-5.21, p=0.001) | 1.37 (0.38-5.03, p=0.630) | 0.94 (0.28-3.13, p=0.923) | - | 3.52 (1.80-6.90, p<0.001) | 1.44 (0.32-6.50, p=0.640) | 1.86 (0.53-6.53, p=0.334) | 1.44 (0.29-7.23, p=0.658) | - | - | - | - |
| Fracture | 1.11 (0.27-4.50, p=0.880) | - |  | - | 0.76 (0.11-5.23, p=0.780) | - | - | - | 0.74 (0.10-5.49, p=0.770) | - | - | - |
| Cancer | 0.97 (0.35-2.71, p=0.950) | - | 0.83 (0.20-3.50, p=0.796) | - | 1.16 (0.35-3.81, p=0.810) | - | - | - | 0.90 (0.28-2.94, p=0.870) | - | 0.44 (0.06-3.25, p=0.417) | 4.77 (0.55-41.49, p=0.157) |
| Stomach ulcer | 1.01 (0.31-3.27, p=0.990) | - | 0.88 (0.12-6.42, p=0.898) | - | 0.92 (0.22-3.78, p=0.900) | - | - | - | 1.25 (0.39-4.05, p=0.710) | - | 1.05 (0.14-7.73, p=0.960) | 0.99 (0.12-8.36, p=0.993) |
| Depression | 1.33 (0.71-2.50, p=0.370) | 1.26 (0.28-5.61, p=0.760) | 1.53 (0.54-4.37, p=0.427) | 3.30 (0.29-37.41, p=0.340) | 1.30 (0.62-2.73, p=0.480) | 0.97 (0.12-7.90, p=0.980) | 1.31 (0.30-5.71, p=0.722) | 1.64 (0.16-17.12, p=0.678) | 1.11 (0.54-2.29, p=0.780) | 1.85 (0.40-8.55, p=0.430) | 1.90 (0.66-5.50, p=0.238) | 2.86 (0.74-11.01, p=0.126) |
| Hypertension_all records | 2.46 (1.52-3.99, p<0.001) | 1.74 (0.74-4.09, p=0.200) | 1.78 (0.90-3.51, p=0.098) | 4.33 (0.54-34.76, p=0.170) | 2.96 (1.70-5.14, p<0.001) | 1.43 (0.49-4.15, p=0.510) | 1.75 (0.73-4.22, p=0.210) | 1.95 (0.60-6.40, p=0.269) | 3.02 (1.77-5.14, p<0.001) | 2.01 (0.71-5.69, p=0.190) | 1.60 (0.76-3.38, p=0.220) | 1.72 (0.70-4.24, p=0.239) |
| Hypertension_baseline | 2.14 (1.31-3.50, p=0.003) | 1.20 (0.50-2.87, p=0.680) | 1.49 (0.74-3.03, p=0.266) | 2.15 (0.43-10.74, p=0.350) | 2.69 (1.54-4.72, p=0.001) | 1.78 (0.63-5.07, p=0.280) | 1.69 (0.69-4.13, p=0.248) | 1.85 (0.58-5.86, p=0.296) | 2.39 (1.38-4.14, p=0.002) | 1.51 (0.57-3.99, p=0.400) | 1.23 (0.55-2.72, p=0.613) | 1.56 (0.61-3.97, p=0.355) |
| Diabetes_all records | 2.60 (1.59-4.26, p<0.001) | 1.74 (0.63-4.74, p=0.280) | 1.07 (0.41-2.77, p=0.887) | 1.78 (0.20-16.08, p=0.610) | 2.58 (1.44-4.63, p=0.002) | 1.80 (0.50-6.52, p=0.370) | 1.57 (0.52-4.69, p=0.423) | 0.54 (0.14-2.09, p=0.371) | 2.23 (1.24-4.04, p=0.008) | 1.64 (0.50-5.44, p=0.420) | 0.87 (0.26-2.86, p=0.814) | 0.61 (0.14-2.67, p=0.508) |
| Diabetes_baseline | 2.41 (1.45-3.99, p=0.001) | 1.80 (0.66-4.94, p=0.250) | 1.12 (0.43-2.88, p=0.822) | 1.85 (0.21-16.61, p=0.580) | 2.46 (1.35-4.49, p=0.003) | 1.88 (0.52-6.82, p=0.340) | 1.64 (0.55-4.91, p=0.378) | 0.54 (0.14-2.09, p=0.371) | 1.96 (1.05-3.66, p=0.035) | 1.74 (0.53-5.78, p=0.360) | 0.91 (0.28-3.01, p=0.880) | 0.61 (0.14-2.67, p=0.508) |
| Start corticosteroids | 1.02 (0.69-1.49, p=0.940) | 0.83 (0.37-1.87, p=0.650) | 1.09 (0.55-2.15, p=0.806) | 0.66 (0.08-5.67, p=0.700) | 1.07 (0.68-1.69, p=0.760) | 1.11 (0.38-3.25, p=0.850) | 2.00 (0.72-5.51, p=0.182) | 4.27 (0.87-20.84, p=0.073) | 0.94 (0.62-1.43, p=0.770) | 1.19 (0.44-3.21, p=0.730) | 0.83 (0.40-1.72, p=0.619) | 1.31 (0.53-3.26, p=0.557) |
